# Influence of *α*-Fe_2_O_3_, CuO and GO 2D nano-fillers on the structure, physical properties and antifungal activity of Na-CMC–PAAm blend

**DOI:** 10.1038/s41598-023-39056-y

**Published:** 2023-07-31

**Authors:** A. Abou Elfadl, Asmaa M. M. Ibrahim, Adel M. El Sayed, S. Saber, Sameh Elnaggar, Ibrahim M. Ibrahim

**Affiliations:** 1 Physics Department, Faculty of Science, Fayoum University, El Fayoum, 63514 Egypt; 2 Department of Botany, Faculty of Agriculture, Fayoum University, El Fayoum, 63514 Egypt; 3 Department of Agricultural Microbiology, Faculty of Agriculture, Fayoum University, El Fayoum, 63514 Egypt

**Keywords:** Biomaterials, Condensed-matter physics, Nanoscale materials, Structural materials, Materials science, Nanoscience and technology, Physics

## Abstract

The present work aims to improve the uses of the carboxymethyl cellulose–polyacrylamide (Na-CMC–PAAm) blend for energy storage, optoelectronic applications, biological control, and plant disease management. Nano-sized materials (*α*-Fe_2_O_3_ nanoplates (NP), CuO NP, and GO nanosheets (NS), were synthesized and incorporated into the blend. The phase purity and morphologies of the used fillers were studied by XRD and HR-TEM. The interactions and complexation between the nano-fillers and the blend chains were investigated using XRD and FTIR spectra. The chemical composition and surface morphology of the nanocomposites were studied using EDS and FE-SEM analysis. UV-vis-NIR spectra revealed that the blend shows about 95*%* transmittance, reduced by 10–30*%* after doping. The absorption and refractive indices, as well as the optical gaps of the blend, were greatly affected by the doping. The dielectric constant and loss depend on the type of filler and the applied frequency. The maximum ac conductivity of the blend at 303 K and 4.0 MHz is 21.5 × 10^–4^ S/m and increased to 23.5 × 10^–4^ S/m after doping with CuO NP. The thermal stability, activation energy, stress–strain curves, and tensile strength are dependent on the filler type. All nanocomposite solutions except the blend exhibited a wide range of antifungal properties against pre- and post-harvest phytopathogenic fungi. *Aspergillus niger* among the examined fungi showed high sensitivity to the tested nanocomposite solutions. Furthermore, the CuO/blend nanocomposite had the highest antifungal activity against all tested fungi. Based on that, we suggest the use of CuO/blend and GO/blend nanocomposites to control and combat pre- and post-harvest fungal plant diseases.

## Introduction

Combining two or more different polymers is a practical and easy method to create blends with unique physical properties. This approach is gaining increasing attention due to the expected new uses of the produced materials as well as its contribution to the basic sciences^1–6^. The sodium salt Na-CMC is a semicrystalline, water-solvent, edible and anionic polymer. Besides its availability and low toxicity, the existence of many COOH/OH groups in its structure creates wonderful coordination interactions with metal ions^7^. Compared with other bio-polymers, Na-CMC is preferred due to its flocculating property, viscosity, and transparency. Na-CMC has higher tensile strength and lower elongation at break compared with starch. In addition, its tensile strength is lower than that of both chitosan and sodium alginate, and its elongation at break is in between the two^8^. Na-CMC has attracted the attention of research groups due to its interesting features needed for several applications in the food industry, *i.e.*, food packaging, drug delivery, tissue engineering, etc.)^9^. Its biodegradability and biocompatibility are essential for the recycling of food waste, sustainability, and enhancing the shelf life of food products^8^.

Similarly, PAAm is a linear, hydrophilic, non-toxic, amorphous polymer with (–CONH_2_) groups. These properties offer a wide range of practical uses for food packaging, waste-water purification, and 3D printing applications. However, due to its weak mechanical properties, some researchers aimed to blend PAAm with Na-CMC to improve its mechanical strength and film-forming ability^7,10–12^.

Based on the good miscibility and common characteristics, several research groups have reported the preparation of Na-CMC–PAAm blend and studied the effect of various nanofillers on the physicochemical and biological properties of this blend. The –CONH_2_ group can create interactions with the drug molecules and therefore facilitate the use of PAAm for drug release and diffusion, and some other medical and pharmaceutical applications^6^. Morsi et al.^7^ developed Li_4_Ti_5_O_12_/Na-CMC–PAAm nanocomposites for energy storage and microelectronic devices, tunable nano-dielectrics, and solid polymer electrolytes for Li batteries. The blend composed of Li polyacrylate and PAAm achieved an ionic conductivity of (13.8 ± 2.4) × 10^–5^ S/m, and a high over-potential in the water decomposition reactions, which proved to be useful for solid-state electrochemical capacitors^13^. Nascimento et al.^14^ applied the free radical polymerization route to prepare Na-CMC–PAAm–cloisite-Na^+^ nanoclay hydrogel with a very porous structure for use as a carrier vehicle for the controlled release of agrochemicals. Yadava et al.^15^ prepared a GO/CMC/alginate composite and found that the incorporation of 1 wt% GO improved the tensile strength and the Young modulus by 40% and 1128%, respectively.

The nano-sized oxides are a very interesting class of materials used as fillers in polymer nanocomposite fabrication. Hematite (*α*-Fe_2_O_3_) is one of the most widely used oxides in various technological fields, including recording devices, rechargeable lithium-ion batteries, biomedical materials, solar cells, and gas sensors. This is attributed to its eco-friendliness, high thermal stability, melting point and resistance to corrosion, and the high theoretical specific capacitance of ~ 3625 F/g. In addition, *α*-Fe_2_O_3_ has a direct band gap of *E*_gd_ = 2.0–2.2 eV and can absorb ~ 40*%* of the solar spectrum^16^. In addition, CuO is a semiconductor with an *E*_g_ in the range of 1.2–2.1 eV and has many advantages, such as a low production cost, non-toxicity, good electronic/thermal properties, and a high dielectric constant of 18.1. Therefore, nanosized CuO is widely used in supercapacitors, solar energy applications, spintronics, high *T*_C_ superconductors, and heat transfer applications^17^. Graphene oxide (GO) NS is an interesting additive to the polymers due to the improved surface area. Its sheet structure is enriched with oxygen- containing groups (–OH, –O–, and –COOH) which are very important for the synthesis of GO/blend films^18^. In addition, these groups on the edges and basal plane lead to relatively high proton conductivity^19^, and therefore GO is an interesting material for fuel cell applications and proton exchange membranes. Zhang et al.^10^ fabricated GO/Na-CMC–PAAm hydrogel with improved compressive strength of 2.87 MPa and enhanced swelling behaviors useful for bioengineering applications and drug delivery systems. According to Madih et al.^19^, the introduction of GO onto cellulose acetate (CA) creates more ionic channels and improves the ionic motion inside the matrix, and enhances the operation of the fuel cells. In addition, GO NS represents an interesting reagent for cancer photo-thermal therapy due to its outstanding near-infrared (NIR) absorbance capability^20^.

In addition to the abovementioned use of nanomaterials as fillers to improve the physicochemical properties of the polymers, they also enhance their antimicrobial properties^21^. The molecules of the nano-sized materials can penetrate the microbial cell walls, produce free radicals, and induce oxidative stress, which irreversibly damages the cellular components of pathogens^2,22^**.** This inhibit fungal spore germination, and gene and protein regulation^22^, leading to microbial cell death. GO's mode of action involves physical interaction with the cell membrane, inducing membrane disruption, and forming GO-cell aggregates^23^. While antimicrobial CuO NPs mechanisms are represented in lipid peroxidation, damage of DNA, membrane, and mitochondria, oxidative stress, and possible meta-ion leaching and dissolution^24^. On the other hand, when Fe_2_O_3_ NPS can generate reactive oxygen species (ROS) inside the microbial cell^25^.

Worldwide, fungal diseases are widely underestimated, despite posing a significant threat to a variety of plant and animal species, as well as public health^26^. In recent decades, the rise in the global population has increased agricultural demand. As a result, there has been global pressure to find ways to improve agricultural crop quality and productivity^26^. The number of virulent phytopathogenic fungi is constantly increasing, resulting in significant economic losses around the world. El-Baky and Amara^27^ stated that there has been a significant increase in the development of fungal resistance to these fungicides, as well as the negative effects of synthetic fungicides on the health of humans, animals, and the environment.

*Penicillium digitatum* and *A. niger* are the most devastating pathogens of apples, pears, peaches, citrus, grapes, figs, strawberries, mangoes, tomatoes, and melons and some vegetables, especially onions and garlic, being responsible for about 90*%* of production losses during post-harvest handling^28^. *A. niger* can produce aflatoxins, ochratoxin A in stored products, which seems very inevitable^29^. *Fusarium oxysporum, Fusarium solani* and* Botryodiplodia theobromae* are types of common soil-borne fungi that lead to vascular wilt and crown, stem, or root rot across a broad host range of economically important crops such as citrus, vegetables, flowers, and field crops worldwide, with a great overall impact on productivity^30^. The utilization of nanomaterials became an interesting strategy for combating plant pathogens^22^. This is owing to their unique physicochemical, and biological properties and their potential for a variety of fields, including the control of plant pathogens and the unambiguous solution to the issues with disease management that can be provided by nanotechnology^31^. Due to their excellent efficacy against a wide spectrum of microbes, CuO and Cu_2_O are among the most extensively used antimicrobial agents^32^. GO is a newly emerging and highly promising antimicrobial agent against various plant pathogens in agricultural science^33^. GO can strongly inhibit the mycelial growth and spore germination of various plant fungal pathogens, such as *Fusarium poaea*, *Fusarium graminearum,* and* F. oxysporum*^34^. Moreover, Sawangphruk et al.^35^ reported a superior antifungal activity of GO NS towards *A. niger*, *Aspergillus oryzae*, and *F. oxysporum*. Also, the nano-composite GO/chitosan exhibited antifungal activity against *Aspergillus nigar* ATCC 9029 and *Penicillium roqueforti*^36^. Moreover, nano-sized *α*-Fe_2_O_3_ has significant antifungal activity against many different fungal pathogens^37^. Saied et al.^38^ reported that hematite NP showed antifungal potential against *Aspergillus fumigatus* and *Candida albicans* with MICs values were 62.5 and 2000 µg/mL, respectively. Muhamad and others^39^ referred that Na-CMC–PAAm hydrogel has a low antimicrobial activity.

Antifungal agents have been widely used as an alternative approach for managing fungal plant diseases^26^. Therefore, there is an urgent need to develop highly effective and safe new antifungals, especially, due to the excessive use of antifungals that are harmful to health and the environment^26^.

The literature survey is almost free of reports on the influence of the above-mentioned oxides on the physical properties of Na-CMC–PAAm blends. Therefore, this work focuses on the fabrication of *α*-Fe_2_O_3_ NP, GO NS, and CuO NP materials as nano-sized fillers to affect the physical and chemical properties of the Na-CMC–PAAm blend. Several characterization techniques have been used to study the structural, optical, electrical, mechanical, and thermal properties of the blend as well as the resistance against the fungus activity.

## Experimental details

### Materials and preparation

Ferric chloride hexahydrate [FeCl_3_.6H_2_O, M_W_ = 270.30 g/mol and purity ~ 98*%*, from Nova Oleochem Limited] and copper (II) acetate [Cu(CH_3_COO)_2_.H_2_O, M_W_ = 199.65 g/mole and purity ~ 98*%*, from Barcelona, PRS Panreac] and oxalic acid [H_2_C_2_O_4_·2H_2_O, M_W_ = 126.07, from LOBA Chemie, India] were used for *α*-Fe_2_O_3_ and CuO nanoplates (NP) fabrications. GO nanosheets (GO NS) were supplied by the Materials Science and Nanotechnology Department, Faculty of Postgraduate Studies for Advanced Sciences, Beni-Suef University, Egypt. PAAm powder, M_W_ = 2.1 × 10^5^ g/mol, from Tianjin Fu Chen Chemical Reagents Factory, China and Na-CMC powder, from El-Nasr Pharmaceutical Chemicals Co., were used for blend preparation. Double-distilled water (DDW) was used as a common solvent. Firstly, 0.7 M FeCl_3_.6H_2_O and Cu(CH_3_COO)_2_.H_2_O solutions were prepared by dissolving 18.92 g and 13.98 g of FeCl_3_.6H_2_O and Cu(CH_3_COO)_2_.H_2_O each in 100 ml DDW using the magnetic stirrer for 1.0 h at room temperature (RT). To each of these solutions, 17.65 g of oxalic acid was added to chelate the Fe or Cu ions (the molar ratios of oxalic acid to FeCl_3_.6H_2_O or Cu(CH_3_COO)_2_.H_2_O is 2:1) after that the stirring process continued for another 1.0 h. The solutions aged for about 24 h at RT. Finally, the excess of water was evaporated from the solutions at 100 °C for 6.0 h and then the samples were calcined at 500 °C in the air using an electric furnace for 2.0 h. The prepared *α*-Fe_2_O_3_ and CuO NP and the supplied GO NS were kept in a zipper bag to avoid the moisture effect. Na-CMC (80*%*)–PAAm (20*%*) blend was prepared by dissolving 0.8 g of Na-CMC in 80 ml DDW by magnetic stirring for 2.0 h at 75–80 °C and dissolving 0.2 g of PAAm in 20 ml DDW by stirring for 2.0 h at RT. Then Na-CMC and PAAm solutions were mixed under stirring for another 1.0 h at ~ 35 °C. The composite solutions were prepared by the ultrasonic dispersion in of the required quantity of the nano-fillers in 20 ml DDL, determined using the equation:

1 $$x (\mathrm{wt}.\mathrm{\%})=\frac{{w}_{filler}}{{w}_{filler}+1}$$

The $x$ was chosen to be 0.5 wt.% only to avoid particle agglomeration inside the blend, “1” in the denominator is the total weight of the polymers, and ${w}_{filler}$ is the weight (~ 0.005 g) of *α*-Fe_2_O_3_ NP, CuO NP and GO NS. The nano-filler solution were added drop by drop under vigorous stirring to the blend solution. The blend and composite solutions were cast in pre-cleaned glass Petri dishes and dried slowly at 40 °C for three days.

### Characterization and measurements

The X-ray patterns of *α*-Fe_2_O_3_, CuO, GO, and the polymeric films were recorded by PANalytical X’Pert Pro X-ray diffraction system utilizing Cu*-K*_α_ radiation of 1.540 Å wavelength. The device operated at 30 kV, and scans were carried out over a range of 5°–80°. The average crystallite sizes (*D*_av_) were calculated utilizing the Scherrer's equation:

2 $$D= \frac{0.9\lambda }{\beta \mathrm{cos}\left(\theta \right)}$$

where *λ* = 0.154 nm is the applied wavelength of X-ray, *θ* is the Bragg’s angle and *β* is the full-width at half maximum intensity. A high-resolution transmission electron microscope (HR-TEM), JEM 2100, Jeol was used to check the size and morphology of the sol–gel prepared *α*-Fe_2_O_3_ and CuO. Fourier transform infrared (FT-IR) spectra were recorded in the 400–4000 cm^−1^ wavenumber range using a Bruker spectrophotometer vertex/70 coupled with a diamond attenuated total reflection (ATR) unit. The films’ surface morphology and chemical analysis were studied using scanning electron microscopy (SEM; Inspect S, FEI, Netherlands), coupled with (energy-dispersive spectroscopy, EDS) unit operating with a 20 kV *e*-beam. Optical spectra were recorded using a Shimadzu UV-3600/UV-VIS-NIR spectrophotometer of ± 0.2 nm accuracy, in the wavelength range of 200–1500 nm. The absorption index (*k*) is related to the absorption coefficient (*α*) by the relation: $k=\frac{\alpha \lambda }{4\pi }$, where $\alpha =2.303\frac{A}{d}$, *A* is the recorded absorption spectra, and *d* is the thickness, that was measured by a digital micrometer and listed in Table S1.The ac conductivity, the dielectric constant *ε'* and dielectric loss *ε''* were measured (utilizing a HIOKI IM3536 LCR meter, with an accurate capacitance measurement in the order of 1 × 10^–4^ pF) in the frequency range of 0.1 kHz–4 MHz and the temperature range of 30–100 °C. PerkinElmer STA 6000 equipment was used to perform thermogravimetry analysis (TGA). The measurements proceeded in the temperature range of 25–550 °C with a heating rate of 10 °C min^−1^ under a nitrogen atmosphere. The activation energy, *E*_a_, of the major degradation process was estimated by using Coats–Redfern’s formula^40^,

3 $$\mathrm{ln}\left[\frac{-\mathrm{ln}(1-\alpha )}{{T}^{2}}\right]=-\frac{{E}_{a}}{RT}+\mathrm{ln}\left[\frac{AR}{\beta {E}_{a}}\left(1-\frac{2RT}{{E}_{a}}\right)\right]$$

where *R*, *A*, *T*, *t*, *β* (*β* = dT/dt) are the universal gas constant (8.314 J/mol K), a constant, the absolute temperature (K), the time (min), and the heating rate, respectively. *α* ($\alpha =\frac{{m}_{i}-{m}_{T}}{{m}_{i}-{m}_{f}}$ ; m_i_ and m_f_ are the initial and the final mass of the decomposition process, respectively. m_T_ is the mass at temperature T) is the fraction of decomposition. For 2*RT*/*E*_a_ <  < 1, the second term in the right side of Eq. 3 is simplified to $\mathrm{ln}\left[\frac{AR}{\beta {E}_{a}}\right]$. Plotting the left side of Eq. 3 against 1/T yields a straight line. The *E*_a_ value can be estimated from the slope of the straight line, *E*_a_ = –slope × R^41^. Stress–strain curves were recorded using a ZwickRoell testing machine, model Z010 TN-Germany, equipped with a 1 kN Load cell. For tests, the prepared films were cut in rectangular form with average dimensions of 8 cm length and 1.5 cm width. The tensile test was replicated three times for each sample at a rate of 50 mm min^-1^ at RT. The average values of measurements were considered.

## Determination of antifungal activity

### Media and fungal strains

The antifungal activity of Na-CMC-PAAm (blend), GO/blend, *α*-Fe_2_O_3_/blend and CuO/blend solutions were established against phytopathogenic fungi represented in* P. digitatum, B. theobromae, F. oxysporum, F. solani* and* A. niger.* All these tested microorganism strains were obtained from the culture collection of the department of botany, faculty of agriculture, Fayoum University, Egypt. The strains of fungi were grown on potato dextrose agar medium. The investigated fungi were incubated aerobically at 25–28 °C for 5–7 days.

### Antifungal activity assay

The antifungal activity of the examined phytopathogenic fungi were studied using the agar well diffusion method (AWDM), according to Gonelimali and others^42^. The Fungal spore suspensions of the tested strains was adjusted at a concentration of 10^6^ spores/ml using microscopic direct counting by hemocytometer as described by Zhang and others^43^. A sterile cork borer with a 6 mm diameter was used to make wells in the inoculated and solidified petri dishes. Two hundred μl of tested solutions were poured into the wells. Before the incubation of inoculated petri plates and to allow the examined solutions to properly diffuse, the plates were refrigerated at 4 °C for 4 h, then followed by aerobically incubation at 25–28 °C for 5 days. The antifungal activities of the studied nano-composite materials were evaluated by measuring the inhibition zone diameter (mm). All experiments were conducted in triplicate.

### Minimal inhibitory concentrations (MIC) and minimum Fungicidal concentrations (MFC) determination

Based on the gar well diffusion test findings, which revealed that three of four of tested solution had antifungal activity (as qualitative method), for the quantitative determination of antifungal activity of Na-CMC-PAAm (blend), GO/blend, *α*-Fe_2_O_3_/blend, and CuO/blend, the MIC and MFC test was conducted and calculated using the broth dilution technique^44^. Na-CMC-PAAm (blend), GO/blend, *α*-Fe_2_O_3_/blend and CuO/blend concentrations were gradually reduced by 50% and tested on the studied phytopathogenic fungi. The MIC concentrations of studied nano-composites were determined to be the first tube, which showed no growth. While the MFC concentrations of studied nano-composites were considered to be the first tube, in which the fungi count was 99.9% lower than the initial count fungal spores.

### Statistical analysis

According to a randomized complete block design, the obtained data were analyzed using the InfoStat computer software package (version, 2012). The least significant difference (LSD) as a mean separation test at 0.05 and 0.01 probability levels was used.

## Results and discussion

### The structural characterization

**Figure 1:**
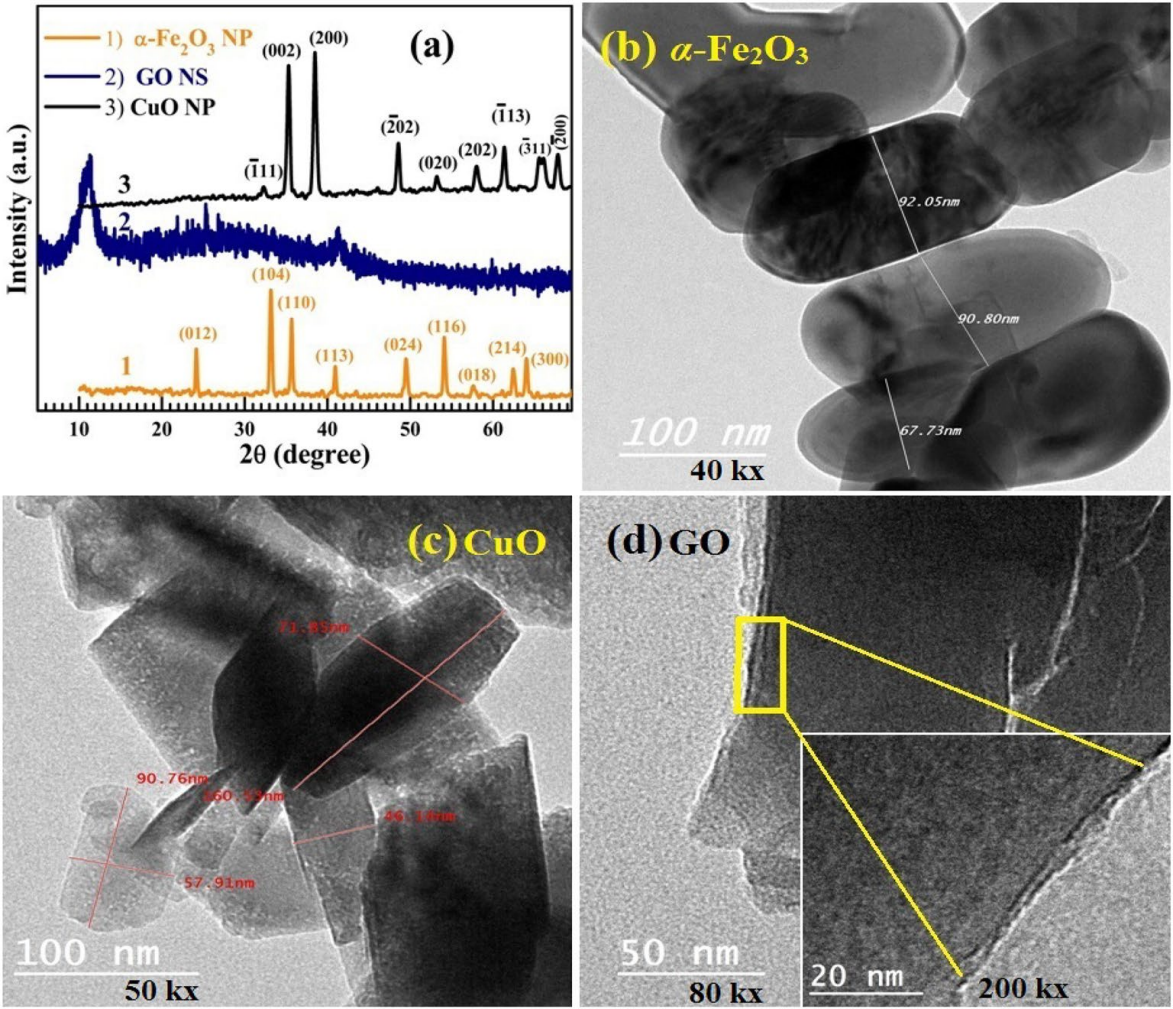
(**a**) XRD patterns of the sol–gel prepared *α*-Fe_2_O_3_, GO and CuO and (**b**)–(**d**) their HR-TEM images. The inset of (**d**) shows NS morphology and layered structure at a higher magnification.

XRD of the sol–gel prepared *α*-Fe_2_O_3_ and CuO and the supplied GO NS are shown in Fig. 1a. In the case of *α*-Fe_2_O_3_; all of the diffraction peaks and their Miller's indices (listed above the peaks in the figure) belong to the rhombohedral/hexagonal structure of hematite with *the R-*3*c* space group. This is in agreement with the 04-015-7029 JCPDS card. No peaks related to other FeO phases or impurities are found in this spectrum, which indicates the high purity of *α*-Fe_2_O_3_. The sharp and strong peaks indicate good crystallinity, where the *D*_av_ was calculated from the most intense peaks, *i.e.*, the (012), (104), (110), and (116) reflections, see the experimental section. The *D*_av_ value was found to be 61.1 nm. XRD spectrum of GO displays an intense peak at 2θ = 10.98°, corresponding to the (001) plane. The wideness of this peak confirms the small *D*_av_ of GO NS. Madih et al.^19^ converted the graphite by the Hummers’ technique (its main peak at 2θ = 26.4°) to GO NS with a characteristic diffraction peak at 2θ = 10.98º. Similarly, Jhang et al.^20^ found that after oxidation of the graphite to GO, the main peak shifted from 26.5° to 10.7° and a small hump/peak around 2θ = 18.5° can be observed. The XRD pattern of CuO contains sharp peaks showing good crystallinity. The diffraction peaks are identified for the monoclinic/tenorite CuO, according to the 45–0937 JCPDS card. Any reflections related to Cu, Cu_2_O, or Cu(OH)_2_ are not detected. The *D*_av_ was found to be ~ 35 nm. Moreover, to investigate the shape and morphology and confirm the size of the sol–gel prepared *α*-Fe_2_O_3_ and CuO powders, HR-TEM images were taken and depicted in Fig. 1 (b-d). As seen, both *α*-Fe_2_O_3_, Fig. 1b and CuO, Fig. 1c, display nanoplate (NP) morphology, 67–92 nm and 47–72 nm wide, respectively, and varied lengths. Whereas, GO displays a layered structure with sheet-like morphology, Fig. 1d.

**Figure 2:**
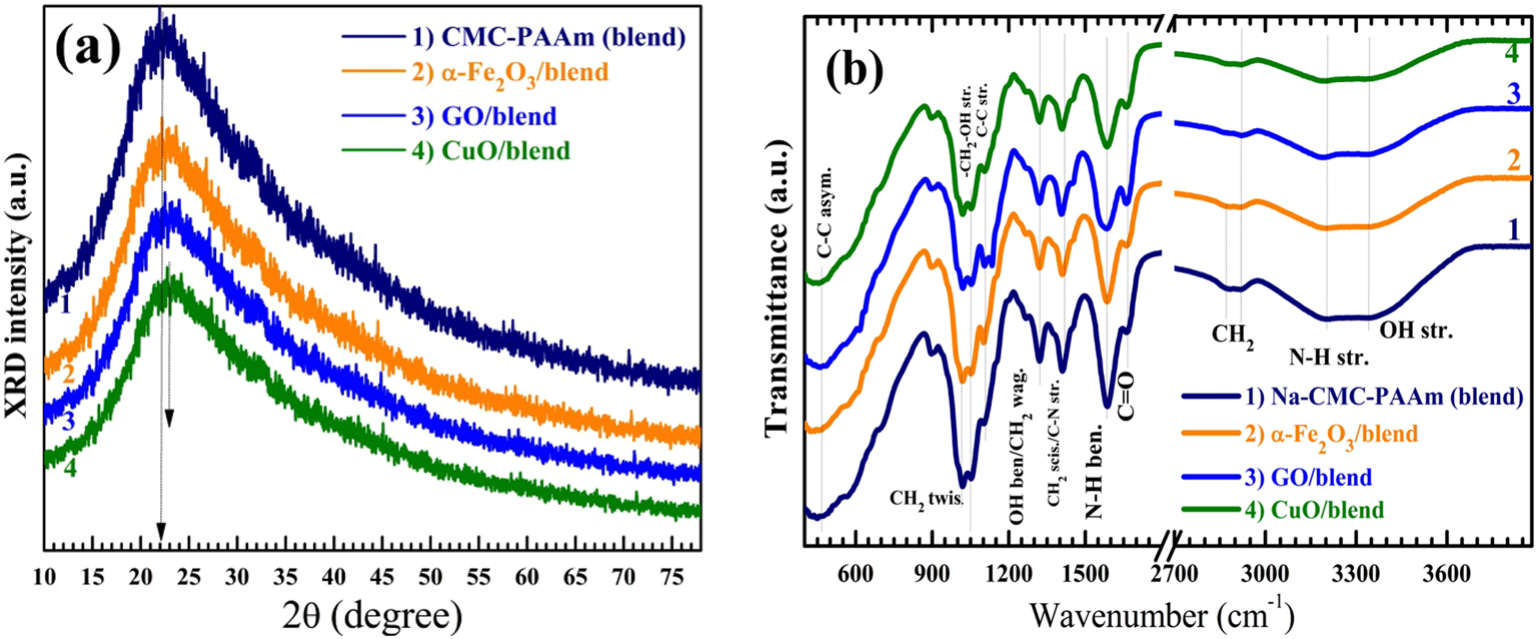
XRD patterns (**a**) and FTIR transmittance spectra (**b**) of Na-CMC (0.8)–PAAm (0.2) blend loaded with different nanofillers.

XRD patterns of the Na-CMC (0.8)–PAAm (0.2) blend, *α*-Fe_2_O_3_ NP/blend, GO/blend, and CuO NP/blend are shown in Fig. 2a. The blend exhibits a semicrystalline nature with a broad peak at 2θ = 22°, where the semicrystalline Na-CMC is the major component. This result agrees well with the literature, where it was reported that the spectrum of Na-CMC has a broad peak around 2θ = 20.7º ^3^. In addition, the pure PAAm has a broad halo around 23°^4^. Similarly, the combination of PVA (2θ = 19.25°) and PAAm (2θ = 21.37°) resulted in a peak for the blend at 2θ = 19.57°^5^. Virya and Lian confirmed the amorphous structure of PAAm where they detected a broad halo in the range of 2θ = 18°–28° in the PAAm spectra^13^. The obtained result concerning the position of the main diffraction peak of our blend indicates the formation of interactions and intermolecular hydrogen bonding between PAAm and Na-CMC chains, which will be discussed in the next paragraph. Moreover, a slight shift can be observed for the position of this main peak after *α*-Fe_2_O_3_ or GO incorporation, and the right shift of ~ 0.74° after adding CuO NP. This shift indicates that these nano-fillers can change the inter-crystalline distance (*x*) due to their interaction and complexation with the functional groups of the blend. It is worth mentioning that the XRD patterns of the nanocomposite samples do not contain any diffraction peaks related to the introduced fillers (*α*-Fe_2_O_3_ NP, GO NS, and CuO NP). In the XRD pattern of CA doped with GO (0.05–0.8 wt%), it was also impossible to identify any diffraction peaks related to GO NS which indicates that the GO was well-dispersed and exfoliated throughout the CA matrix^19^. The *x* value of the blend can be calculated from the position of the main peak maximum using the following equation^2^:

4 $$x= \frac{5\lambda }{8\mathrm{sin}\theta }$$

The *x* value is found to be 0.504 nm, decreased to 0.488 nm after loading CuO NP. This means that the crystalline planes of the blend become closer and the material become more compact. Moreover, the width of this main peak of the blend becomes wider and with less intensity, especially after CuO NP loading, which indicates that the crystallization degree (X_C_*%*) is minimized. Lee et al.^45^, stated that:

5 $${X}_{C}(\%)= \frac{Area\,under\,crystalline\,peak \times 100}{Total\,area\,under\,the\,curve}$$

The calculated X_C_% values are 20.22%, 18.89%, 18.02%, and 17.62% for the blend and the blend loaded with *α*-Fe_2_O_3_, GO, and CuO NP, respectively, see Table S1. This means that the incorporation of these dopants increases the amorphous regions in the blend, yields a more flexible polymer, creates more defects, and enhance the charge carriers formation. This will improve the optical and electrical conductivity of the produced nanocomposites. Loading 2.0% MWCNTs into Na-CMC–PAAm (50%/50%) blend reduced *X*_C_ from 23.01% to 19.03% and increased the free volume, *i.e.*, the open spaces or holes in the blend formed due to the irregular molecular packing in the amorphous regions and the molecular relaxation of the chains^11^. Ragab and Rajeh^5^ reported the decrease of ${X}_{C}$ of Na-CMC–PVA blend from 35.78% to 20.34% after loading 0.8 wt% Ag NP. Morsi et al.^7^ reported the decrease of Xc of Na-CMC (50%)/PAAm (50%) from 24.1% to 17.6% after doping with 0.16 wt% Li_4_Ti_5_O_12_ nanoparticles. According to Al‑Muntaser et al.^46^, Xc of the PVA (75%)/Na-CMC (25%) blend decreased from 21.45% to 13.94% with increasing α-MoO_3_ nanopartilces till 9.0 wt%.

FTIR spectral analysis is performed and depicted in Fig. 2b. The band at 3350 cm^–1^ is due to OH stretching frequencies from the CMC polysaccharide. The peak centered around 3195 cm^–1^ is assigned to the N–H stretch of the amide group. At 2910 cm^–1^ the asymmetric vibration of CH_2_ takes place and the little vibration at 2860 cm^–1^ is due to the symmetric mode of vibration of the same group. The vibration at 1660 cm^–1^ is arising from the stretching of C=O of PAAm in the blend^5,11^. The 1594 cm^–1^ vibration is arising from the amide II^6^. CH_2_ scissoring (CMC)/C–N stretching (PAM) is observed at 1417 cm^–1^. At the energy of 1322 cm^–1^, OH bending (CMC)/CH_2_ wagging (PAM) occurs^6^. The very small peak at 1089 cm^−1^ may be attributed to C–C stretching mode^11^. The primary alcoholic, –CH_2_–OH, stretching modes is detected at 1057 cm^–1^^7^, while the CH_2_ twisting vibration mode is found at 1021 cm^–1^^6^. Finally, at 464 cm^–1^ the asymmetric vibrational modes of C–C occurs^14^. These results confirm the existence of all functional groups of both Na-CMC and PAAm. The presence of the OH group in Na-CMC and NH_2_ in PAAm facilitates the intramolecular interactions and hydrogen bond formation to link the two polymers and the blend chains with the added nanofillers. As seen the intensity of most of the peaks is reduced by a certain ratio depending on the type of fillers. These observed structural variations confirm the random distribution of these nanofillers inside the Na-CMC–PAAm blend. The structural changes, hydrogen bond formation, and complexation induced by the added nanofillers are expected to enhance the optical and electrical properties of the Na-CMC-PAAm blend to make it suitable for a wide range of practical uses and technological applications, as will be discussed in the next sections^11^.

**Figure 3:**
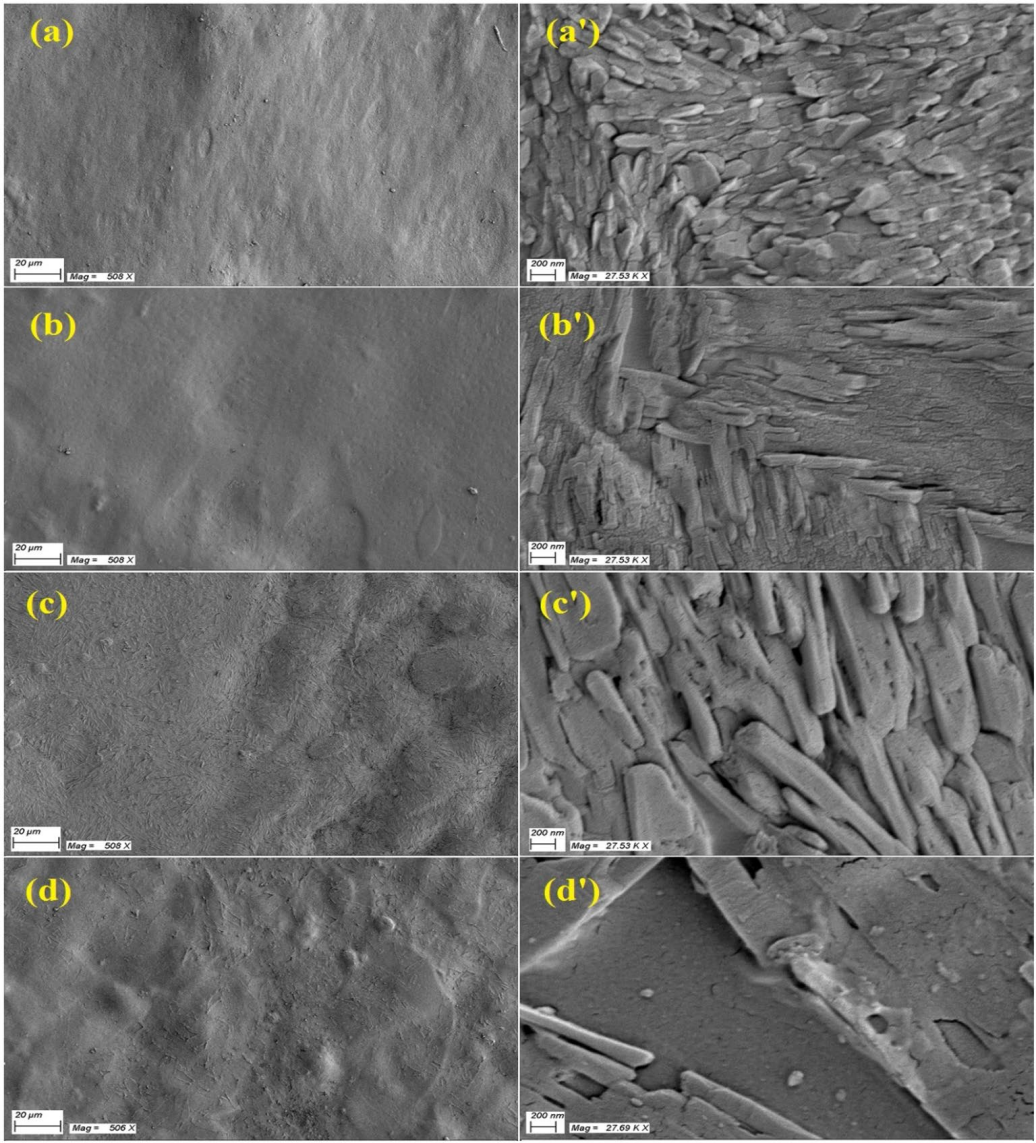
(**a**), (**a'**) SEM image for Na-CMC–PAAm (80%/20%) blend, and the blend loaded with (**b**), (**b'**) *α*-Fe_2_O_3_, (**c**), (**c'**) GO and (d,d') CuO, respectively. The scale bar and magnification are **20 µm** and **508 × **in the left column (**a**)–(**d**) and are **200 nm** and **27.53 kx** in the right column(**a'**)–(**d'**).

Figure 3 shows FE-SEM images for (a) Na-CMC–PAAm blend and (b–d) its nanocomposite films. All films, Fig. 3a–d, are homogenous, and have crack-free surfaces. No phase separation can be observed, indicating the good miscibility of the two polymers (Na-CMC and PAAm). However, particle agglomeration took place in some positions in the blend doped with GO NS, Fig. 3c and CuO NP, Fig. 3d. When we look at the surfaces more closely (magnification 27.53 kx), Fig. 3a'–d', you can see that they have different shapes. For example, the blend surface seems to be made up of a lot of small islands that are randomly arranged on the wall surface. This structure turned out to be a rode-like morphology in the *α*-Fe_2_O_3_/blend, Fig. 3b'. Loading GO NS makes the whole film surface appear to be composed of micro-rods aligned parallel to the surface, Fig. 3c'. CuO NP agglomeration appears clearly at this higher magnification, Fig. 3d'.

The chemical composition analysis was performed and shown in Fig. S1(a-c), see the supplementary materials file. The tables (inset) of this figure show the concentration of each element in the film. The blend is composed of 42.22% C, 46.1% O, 4.97% N, and 6.72% Na coming from the main component of the blend (Na-CMC). A slight variation in the values of these ratios was induced after doping the blend with *α*-Fe_2_O_3_ and CuO NP. Small signals have appeared at 0.705 keV (Fig. S1b) and 0.929 keV (Fig. S1c) coming from Fe *L*_α1_ and Cu *L*_α1_, respectively. EDS is a more sensitive technique, compared with XRD, to detect these small concentrations. The blend and GO/blend showed similar compositions, where GO is composed of C, O, and H (not detectable), and not shown here. Fig. S2 (see the supplementary materials file) shows additional SEM images at a magnification of only 40 x (scale bar 600 µm) used in this investigation. As seen, *α*-Fe_2_O_3_ and CuO NP are distributed all over the films' surface.

### Optical features of Na-CMC–PAAm-based nanocomposites

**Figure 4:**
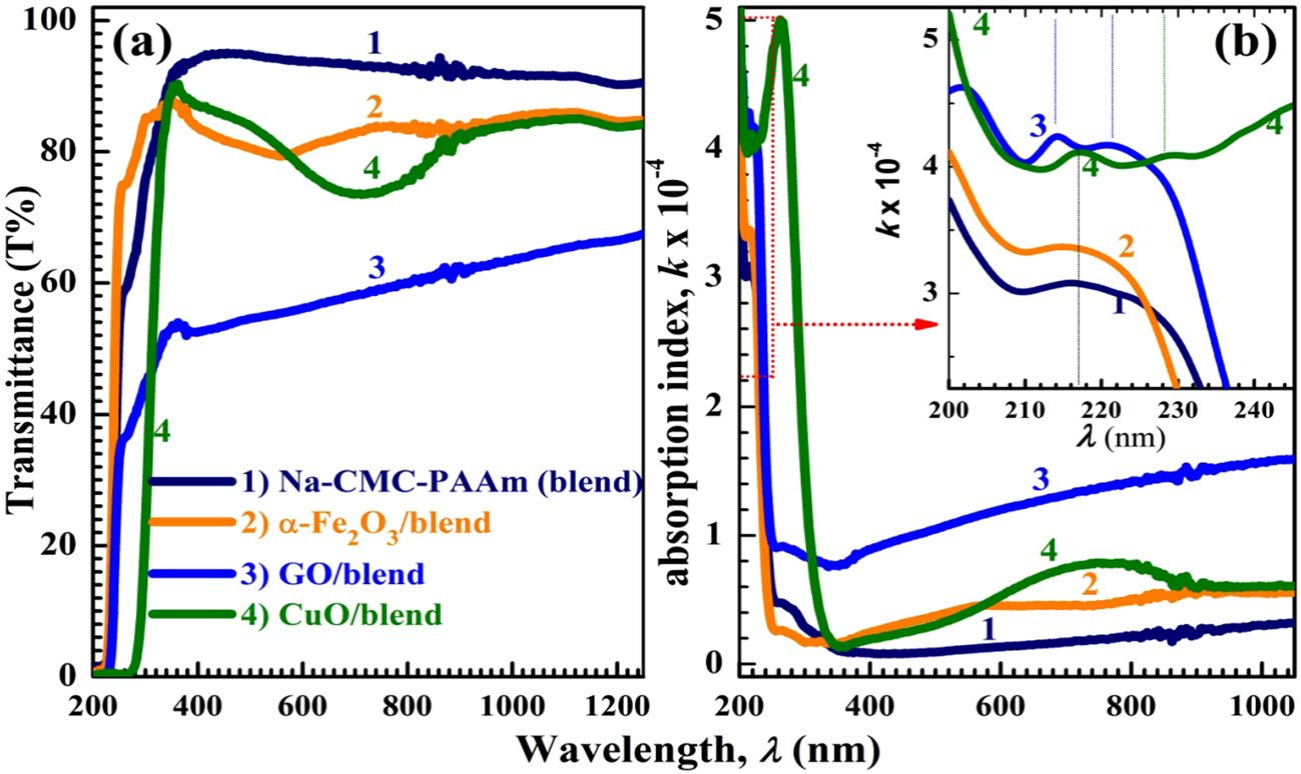
UV/vis transmittance spectra (**a**) and absorption index (**b**) for Na-CMC–PAAm blend and nanocomposites.

Figure 4a shows the transmittance (T%) spectra of the samples. The Na-CMC–PAAm (80%/20%) blend shows a maximum T% of about 94.8*%*. The average T*%* was decreased to around 85% after loading *α*-Fe_2_O_3_ NP and CuO NP. However, after doping with GO NS, T% became in the range of 54–68%, *i.e.*, GO NS are the most fillers capable of hindering/preventing the light to pass through the Na-CMC–PAAm blend. The values of T% at 500 nm wavelength are listed in Table S1. The obtained values of T% are good, where it is known that the T% in 68–92% is enough for many applications^11^. In addition, CuO NP moved the absorption edge significantly to higher wavelengths, indicating a narrowing of the *E*_g_ of the blend. The dependence of the absorption index, *k*, on *λ* of the blend and its nanocomposites is shown in Fig. 4b, where *k* values improved after nanofillers incorporation. However, all films have very low *k*; in the UV region, *k* is ˂ 5 × 10^–4^ and becomes below 1.6 × 10^–4^ in the visible and IR regions. The inset of Fig. 4b exhibits an absorption band at ~ 216 nm for the blend, *α*-Fe_2_O_3_ /blend and CuO/blend and at 214 nm for GO. This band is assigned to *n* → π* electronic transitions because of the presence of unsaturated ethylene and the C = O group in the Na-CMC–PAAm blend which appears at 1660 cm^–1^ as discussed Fig. 2b^7,47^. In addition, GO/blend and CuO/blend also exhibit another small band at ~ 221 and 228 nm, respectively, which may be due to the absorption in GO and CuO compounds, respectively. These bands confirm the interaction and complexation between the added fillers and the functional groups of the blend, as discussed in the FTIR results. The red shift of the absorption edge observed in the CuO/blend spectrum indicates the increase of disordered and amorphous regions after doping with CuO NP^47^, and this finding is consistent with XRD data, see Fig. 2a. Another band at 275 nm in the spectrum of the blend doped with *α*-Fe_2_O_3_ is related to $\pi \to \pi *$ electronic transitions. The curve of CuO/Na-CMC–PAAm blend contains an absorption band (hump) that extends from 600 to 840 nm, which is attributed to $O^{2} \to {\text{Cu}}^{2 + }$ charge transfer^6^.

**Figure 5:**
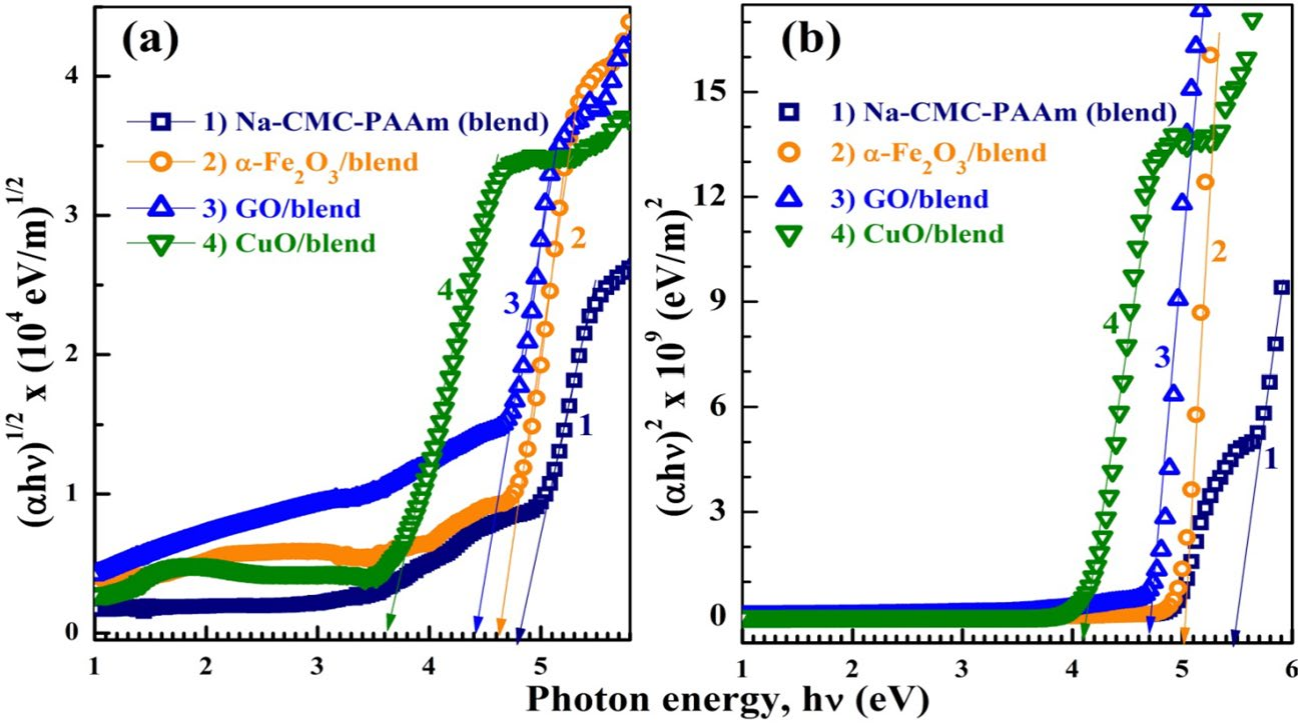
Tauc's relation: (*αhυ*)^1/2^ versus *hυ* and (*αhυ)*^2^ versus *hυ* for indirect (**a**) and direct (**b**) band gap determination, according to Eq. 6.

To determine the values of *E*_g_ of the Na-CMC–PAAm blend and its nanocomposites, we used Davis and Mott relation^48^:

6 $$\left( {\alpha h\upsilon } \right)^{y} \, = \,C\left( {h\upsilon {-}E_{{\text{g}}} } \right)$$

where *C* is a constant,* hυ* (eV) = $\frac{1242}{\lambda (nm)}$ is the incident photon energy, and y = $\frac{1}{2}$ and 2 for the indirect (*E*_gi_) and direct (*E*_gd_) transitions, respectively. The estimated *E*_gi_ and *E*_gd_ values from Fig. 5a,b are listed in Table S1. Incorporation of nanosized materials (NP, NS) inside Na-CMC–PAAm assists the electrons in the valence band to jump to the conduction band via the formation of charge transfer complexes and 3D conductive networks or pathways with the blend chains^47^. As a result, the forming localized states in the forbidden gap minimize the *E*_g_ values of nanocomposite samples. Both *E*_gi_ and *E*_gd_ of our blend decreased from 4.8 and 5.5 eV to 3.6 and 4.1 eV, respectively, due to CuO NP incorporation. The other dopants induced a relatively smaller effect on the *E*_gi_ and *E*_gd_ of the blend. This means that CuO is more effective than *α*-Fe_2_O_3_ and GO in improving the semiconducting properties of the Na-CMC–PAAm blend. In a previous work^11^, the (*E*_gd_ & *E*_gi_) of PAAm, Na-CMC and Na-CMC–PAAm (75*%*/25*%*) blend were found to be (4.98 & 4.60 eV), (4.8 & 4.2 eV) and (5.27 & 4.90 eV), respectively. In addition, 2.0 wt*%* MWCNTs was found to narrow the (*E*_gd_ & *E*_gi_) values of 50%/50% blend from (5.23 & 4.8 eV) to (4.5 & 4.3 eV), which indicates that our 2D nano-filler are more suitable than MWCNTs for band gap reduction. Moreover, Elashmawi and Al. Muntaser reported the decrease of direct and indirect *E*_g_ of Na-CMC (50%)–PAAm (50%) from 5.38 and 4.97 eV to 4.48 and 3.41 eV, respectively, after doping with 0.1 wt.% Co_3_O_4_ nanoparticles^6^.

**Figure 6:**
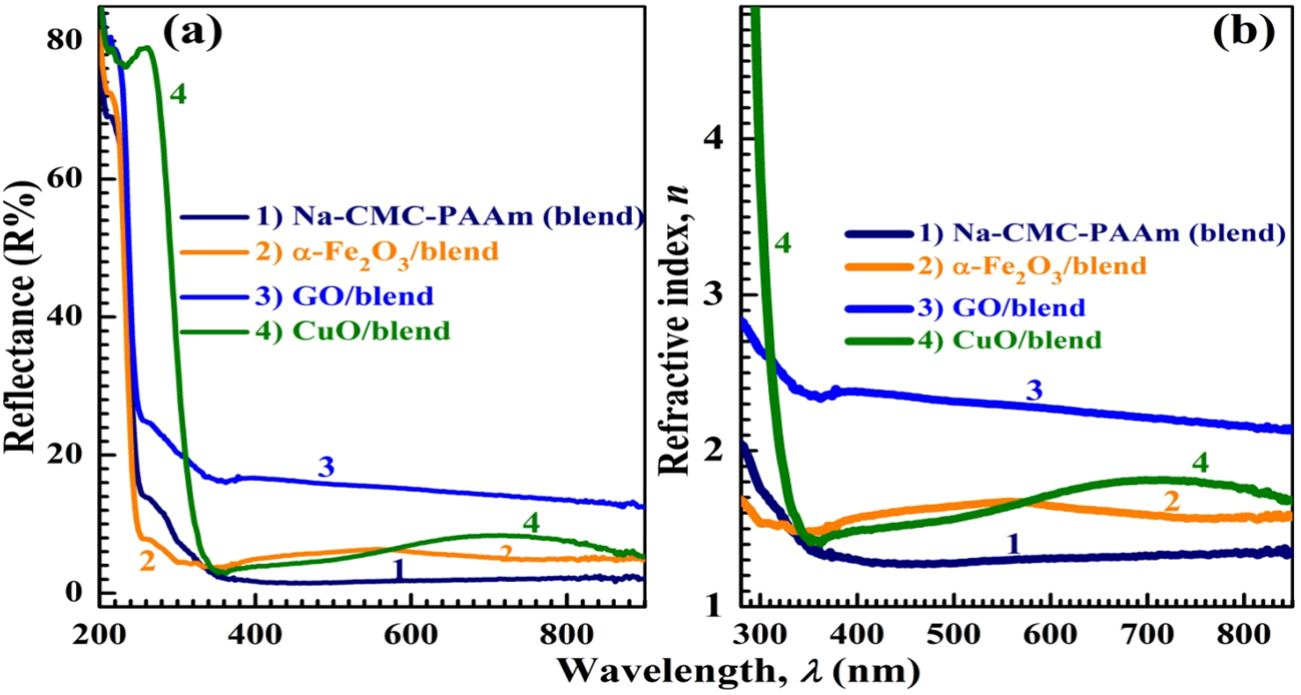
The reflectance spectra (R*%*) (**a**) and the refractive index distribution (**b**) for the blend and nanocomposites.

The index of refraction (*n*) is a very important factor in optical communication for designing optoelectronic devices. One of the simple methods to obtain the n value is by using the reflectance spectra (R*%*)^48^:

7 $$R={\left(\frac{n-1}{n+1}\right)}^{2}\Rightarrow n=\frac{1+\sqrt{R}}{1-\sqrt{R}}$$

where *n* is the real portion of the complex refractive index; $\check{n} = n + ik$. Figure 6a,b shows the R*%* spectra and *n* distribution of the polymeric films. All films exhibit high reflectivity in the UV region. In the visible and IR region, the blend film exhibits very low R slightly enhanced after doping with *α*-Fe_2_O_3_ and CuO NP and becomes in the range of 13–17*%* for GO/blend film. Figure 6b shows that the *n* value Na-CMC–PAAm blend is around 1.35 in the visible and IR regions, which is smaller than the value reported for the blend composed of equal portions from the two polymers^12^. The incorporation of NP inside the polymer blend is expected to improve the n values that already increased after doping with *α*-Fe_2_O_3_ and CuO and *n* are in the range of 2.3–2.7 for GO/blend. The *n* values at 500 nm are inserted in Table S1. In summary, the optical properties of the blend under study significantly improved after loading with the nano-sized fillers. CuO NP/blend is more suitable for photo-catalytic and solar cell applications due to the significant reduction in *E*_g_ of the blend, whereas, GO/blend is more suitable for antireflection coatings due to the significant increase in reflectivity and index of refraction.

### Dielectric characterization of the nanocomposites

**Figure 7:**
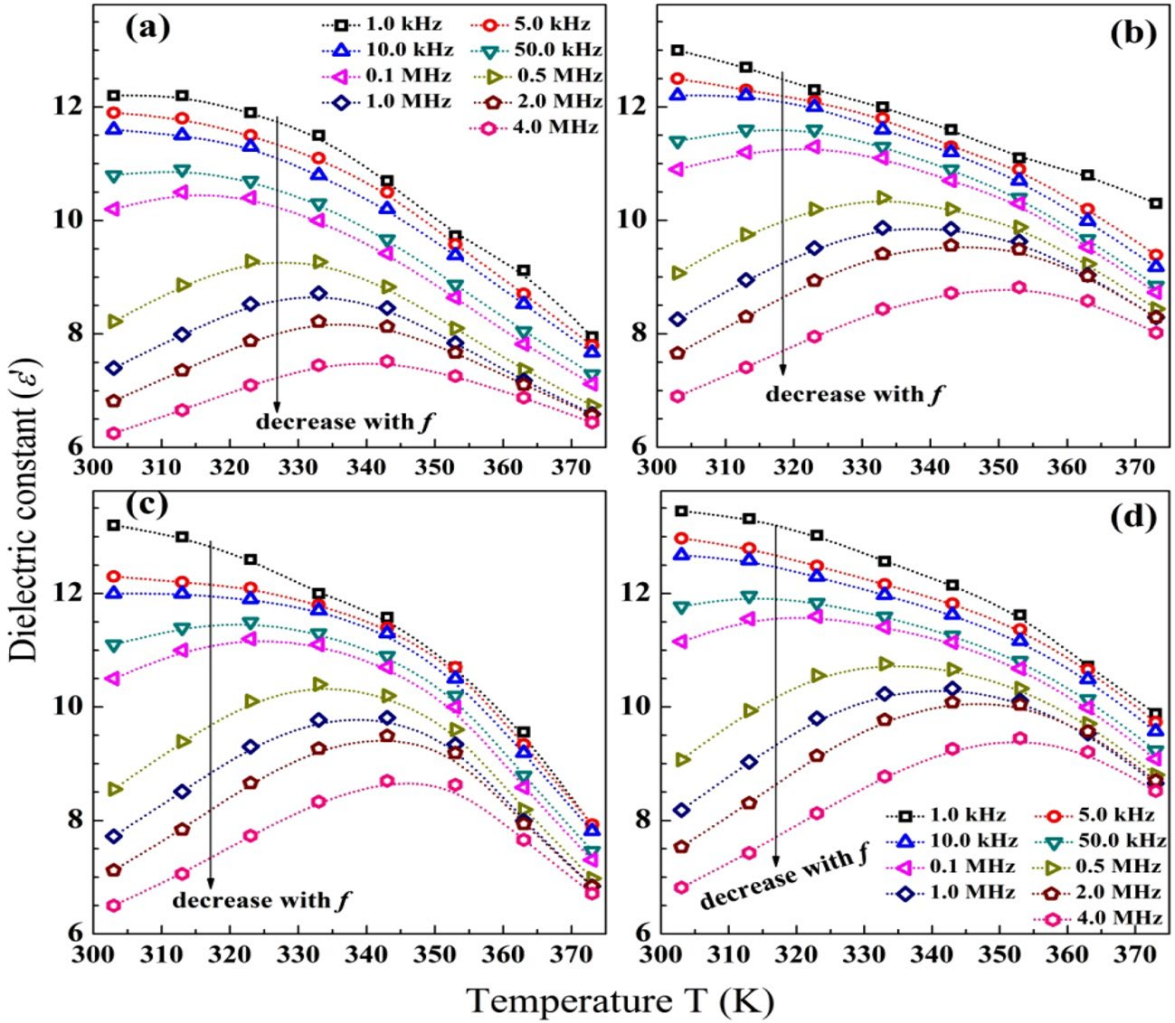
Variation of the dielectric constant with the temperature, at different frequencies, for pure blend (**a**), blend loaded with *α*-Fe_2_O_3_ NP, GO NS and CuO NP (**b**)–(**d**).

Figure 7(a-d) shows the variation of the dielectric constant (*ɛ′*) of Na-CMC–PAAm blend before and after loading *α*-Fe_2_O_3_ NP, GO NS and CuO NP in the temperatures range of 300–375 K, at some selected frequencies in the range of 1.0 kHz–4.0 MHz. The pure blend has *ɛ′* in the range of 6.25–12.5, depending on both *f* and the applied temperature. At any given temperature, *ɛ′* decreases with increasing *f*. In addition, at low temperatures (˂ 325 K) and low frequencies (˂ 10 kHz), *ɛ′* is high and constant. This is because of the accumulation of charge carriers near the electrodes. In other words, the low *f*, a space charge layer is building up in the interface between the sample and the electrode and the charges or dipoles have enough time to accumulate and change their directions according to that of the applied field7^7,46^. As *f* Increases, the period time of the applied field becomes shorter and the dipoles can't reorient fast enough, resulting in a decrease in *ɛ′* values. This means that the contribution dipoles to the polarization becomes smaller^49^. Moreover, at *f* ≥ 0.5 MHz, the *ε'* increases with increasing the temperature until a certain limit thereafter *ε'* decreases with further increase in the temperature, and a relaxation peak (*α–*process) appears with right-shifting to higher temperatures with increasing *f*. This phenomenon means that the dielectric behavior is dipolar^50^. This *α–*process is arising due to the micro-Brownian motion along the main chains in the amorphous regions of the blend.

The nanocomposite films exhibit higher *ε'* values compared with the blend, where at 303 K the *α*-Fe_2_O_3_/blend has *ε'* in the range of ~ 7–13, CuO/blend has *ε'* in the range of ~ 6.75–13.5, and the GO/blend has *ε'* in the range of 6.5–13.25. In addition, these nanocomposites have higher *ε'* values at 373 K compared to the values of the un-doped blend. The incorporation of these 2D nano-fillers introduces heterogeneity inside the blend matrix and hence provides interfacial polarization, which increases the value of *ε'*. Similarly, the heterogeneity created by silver-barium titanate nanofibers increased the εˊ of a polymer from 8.6 to 19.4 after loading^51^. The existence of these 2D nano-fillers may cause a displacement for the O–H and the other anion groups of the Na-CMC–PAAm blend, resulting in the formation of ordered phases inside the amorphous regions in the blend. In addition, doping with the nano-fillers increases the disordered structure and reduces the crystallinity of the blend, as seen in XRD data, and thus the trap centers increase, and the potential barrier to the de-trapping process decrease. In other words, the incorporated nano-fillers may form charge transfers among the blend chains yielding higher polarization^52^.

**Figure 8:**
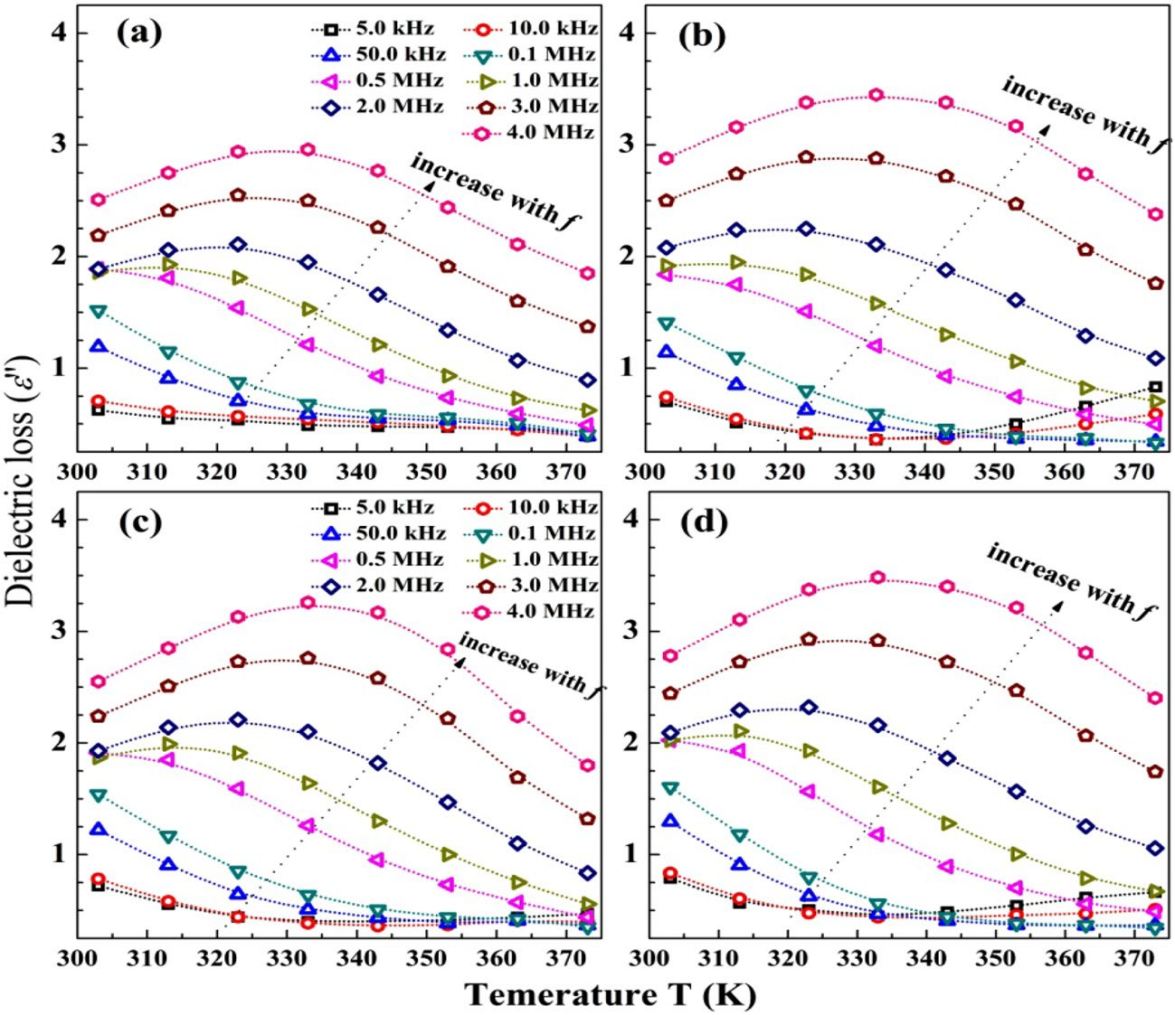
Dependence of the dielectric loss on temperature, at different frequencies, for pure blend (**a**), blend loaded with *α*-Fe_2_O_3_ NP, GO NS and CuO NP (**b**)–(**d**).

Figure 8a–d shows the dependence of the dielectric loss (*ε*'') on the temperature at frequencies in the range of 5.0 kHz–4.0 MHz. The *ε''* is a measure of the energy loss caused by the micro-Brownian motion of the dipoles and depends basically on the system viscosity which influences by the temperature^53^. At the low *f* (*f* ≤ 10.0 kHz), the *ε''* is very small and constant with increasing the temperature for of the blend and GO/blend, but slightly increases at temperature ˃ 343 K in case of *α*-Fe_2_O_3_/blend and CuO/blend. This means that the absorbed thermal energy is small and has no effect on the motion of the blend chains at low frequencies. Increasing the applied *f* till 0.5 MHz results in increasing the *ε''* values which decrease linearly with the temperature. At *f* ≥ 2.0 MHz, the *ε''* increases with temperature and thereafter decreases with a further increase in the temperature. This relaxation peak shifts slightly to a higher temperature with increasing the applied *f*. This peak occurs below the melting temperature of the blend and nanocomposites, and is related to the molecular motion of the main chains, and means that the mobility of long ranges is converted to short ranges^50,52^. The increasing trend of *ε''* with *f* may be due to the increase in the molecular vibrations in the crystalline regions of the Na-CMC–PAAm blend^54^. This behavior confirms that the motion of the molecules/blend chains is strongly dependent on the frequency (which has a decisive effect)^55^. One can note that all films have very similar values of *ε*'' (the ${\varepsilon }_{max}^{{\prime}{\prime}}$ in the range of 3.0–3.5). While loading *α*-Fe_2_O_3_ and CuO increases the *ε',* all the films have nearly constant *ɛ''*. These materials could be tailored for energy storage applications and supercapacitors^51^.

Fig. S3 shows the variation of the ac conductivity (${\sigma }_{ac}=2\pi f{\varepsilon }_{\mathrm{o}}\varepsilon {\prime}{\prime}$, where ${\varepsilon }_{\mathrm{o}}$ is the dielectric permittivity of the air, 8.854 × 10^−12^ F/m) with *f* in the range of 1.0 kHz–4.0 MHz at two different temperatures. The ${\sigma }_{ac}$ increases exponentially with *f* according to Jonscher’s empirical power law ${\sigma }_{ac} \alpha$ ${f}^{s}$, where *s* is a temperature-depended exponent that determines the conduction mechanism. The maximum ${\sigma }_{ac}$ value at (4.0 MHz) increases from 21.5 × 10^–4^ to 25 × 10^–4^ S/m with increasing the temperature of the blend from 303 to 333 K. The corresponding values (at 303 & 333 K) for *α*-Fe_2_O_3_/blend, GO/blend and CuO/blend are (24 × 10^–4^ & 28.5 × 10^–4^ S/m), (22 × 10^–4^ & 29 × 10^–4^ S/m), and (23.5 × 10^–4^ & 30 × 10^–4^ S/m), respectively. This confirms that the incorporation of 2D nano-fillers inside the blend facilitates the charge transfer complexes by forming 3D semiconducting networks among the polymeric chains and between the incorporated ions and the chains^50^. The insets of Fig. S3 show the plots of log *σ*_*ac*_ versus the reciprocal of temperature (1000/T). The overall temperature dependence of conductivity curves can be separated into two regions I and II, with a slight rate of conductivity enhancement in the region I (at 0.5 and 1.0 MHz), due to the available thermal activation of the polymer chains in this region, and a decreasing trend in the conductivity with increasing the temperature in region II. This illustrates that the ${\sigma }_{ac}$ of the blend and nanocomposites follows the Arrhenius relation:

8 $$\sigma ={\sigma }_{o}\mathrm{exp}\left(\frac{{ -E}_{a}}{{k}_{B}T}\right)$$

where *σ*_o_, *E*_a_ and *k*_B_ are a pre-exponential factor, the activation energy, and the Boltzmann constant, respectively.

### Thermal stability of the nanocomposites

**Figure 9:**
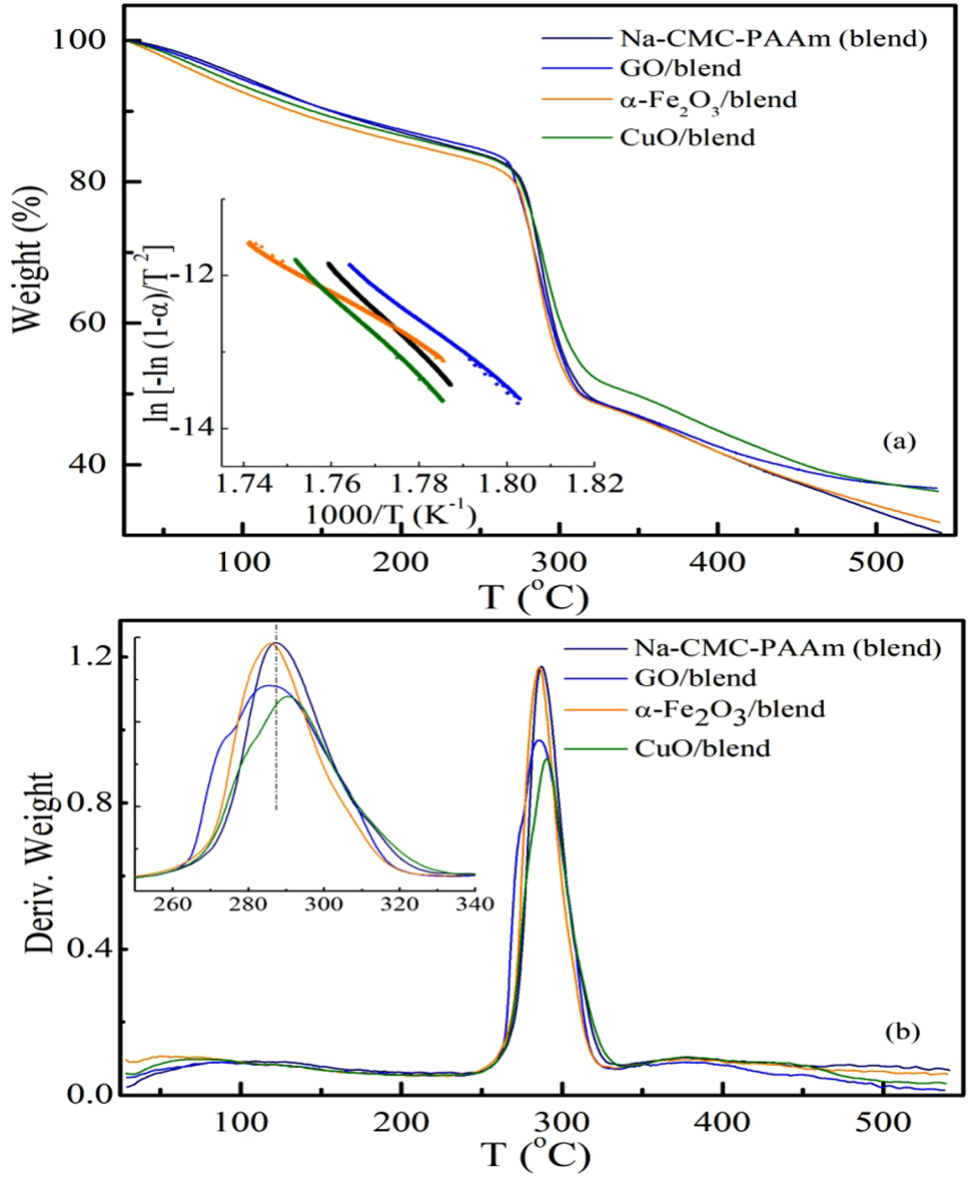
(**a**) TGA thermograms and (**b**) DTG thermograms for Na-CMC–PAAm blend and nanocomposites. The inset of (**a**) shows the temperature dependence of ln [− ln (1 − α)/T^2^] of the 2nd decomposition stage for all samples; solid lines are linear fits. The inset of (**b**) shows a zoomed-in view of DTG in the regime of the main peak.

The influence of *α*-Fe_2_O_3_, GO and CuO on the thermal stability of Na-CMC-PAAm blend was studied by employing the thermogravimetric (TGA). Figure 9 represents the TGA and the corresponding derivative thermogravimetry (DTG, change in mass loss rate) profiles of Na-CMC-PAAm blend and its nanocomposites, namely *α*-Fe_2_O_3_, GO and CuO/ Na-CMC–PAAm blend. The TGA curves present almost two distinct weight loss stages. The first stage at temperatures less than 180 °C with a maximum decomposition temperature at about 90 °C is mainly ascribed to the release of the absorbed water and some light volatile degradation^7,56,57^. It is important to note that the amount of water in all nanocomposites is slightly higher than that of the Na-CMC–PAAm blend where α-Fe_2_O_3_/blend shows the highest of 11.7*%*. The second stage in the range of 240–310 °C is ascribed to the decarboxylation of the COO– functional groups of Na-CMC and pyrolysis of the cellulosic backbone. Adding *α*-Fe_2_O_3_, GO and CuO shifts the initial decomposition temperature *T*_i_ of the Na-CMC–PAAm blend to lower values, Table 1. Above 310 °C, gradual weight losses are noticed for the Na-CMC–PAAm blend and its nanocomposites demonstrating the presence of a different pyrolysis reaction mechanism^58^ and/or the scission of PAAm chains and decomposition of the cyclized imide group^59^. In addition, as shown in Fig. 9a, the residue of *α*-Fe_2_O_3_, GO and CuO/blend nanocomposite films was higher than that of the Na-CMC–PAAm blend. First inspection of the TGA thermograms reveals that the stability of the blend was altered after loading *α*-Fe_2_O_3_, GO and CuO since the peak associated with the greatest decomposition (*T*_m_), given by the DTG, of the blend were reduced from 287 to 285 °C and 286 °C for *α*-Fe_2_O_3_/blend and GO/blend, respectively, and increased to 291 °C for CuO/blend. Thus, although similar behavior is observed for the Na-CMC–PAAm blend and its nanocomposites, CuO/blend nanocomposite shows the highest main decomposition temperature value indicating higher thermal stability. The results suggested that the inclusion of *α*-Fe_2_O_3_ and GO perturbs the van der Waals interaction between the Na-CMC-PAAm chains which depresses its thermal stability^60^, whereas CuO enhances the interfacial interactions between them and thus improves the blend thermal stability.

**Table 1:** Thermal properties of the blend loaded with α-Fe_2_O_3_, GO and CuO in the 2^ed^ decomposition stage: Maximum decomposition temperature (*T*_m_), The initial decomposition temperature (*T*_i_) and the activation energy *E*_a_.

Film composition	*T*_m_ (°C)	*T*_i_ (°C)	*E*_a_ (kJ/mol)
Na-CMC–PAAm (blend)	287	277	491.8
*α*-Fe_2_O_3_ NP/blend	285	274	335.3
GO NS/blend	286	271	411.1
CuO NP/blend	291	276	520.3

The influence of the nanofillers on the thermal stability of the Na-CMC–PAAm blend was further verified by estimating the activation energy, (*E*_a_)of the thermal decomposition (second stage). Generally, in the DGA curves, the complicated degradation process involves several peaks and shoulders, and thus it is hard to determine the peak temperatures *T*_m_ of the decomposition. Applying the combined analysis of DTG and the differential DTG (DDTG) curves allows identification of existence of multiple degradation processes and resolves the strong overlapped peaks. In practice, the maxima at DTG curve, mostly, correspond to the local minima at DDTG curve. Therefore, the combined DTG and DDTG curves were used to locate the peak temperature and ascertain the temperature window of the considered degradation stage (figure not shown). Based on Coats–Redfern’s method (cf. Eq 3), the temperature dependence of ln [− ln (1 − α)/T^2^] is plotted in the inset of Fig. 9a for all samples. Fitting the data to a straight line, the *E*_a_ value was estimated from the slope and inserted in Table 1.

The results reveal that the *E*_a_ value of the Na-CMC–PAAm blend increased from 491.8 to 520.3 kJ/mol upon loading with CuO, whereas the addition of *α*-Fe_2_O_3_ and GO decreased its value to 335.3 and 411.1 kJ/mol, respectively. The higher activation energy of CuO/blend confirms its higher thermal stability relative to the others. The lower *E*_a_ values for *α*-Fe_2_O_3_/blend and GO/blend may be due to the increase of amorphous region as illustrated in XRD results. On the other hand, the higher value for CuO /blend could be ascribed to its denser internal structures which is associated with smaller *x* value (see XRD results)^1,61^.

### Mechanical properties of the nanocomposites

**Figure 10:**
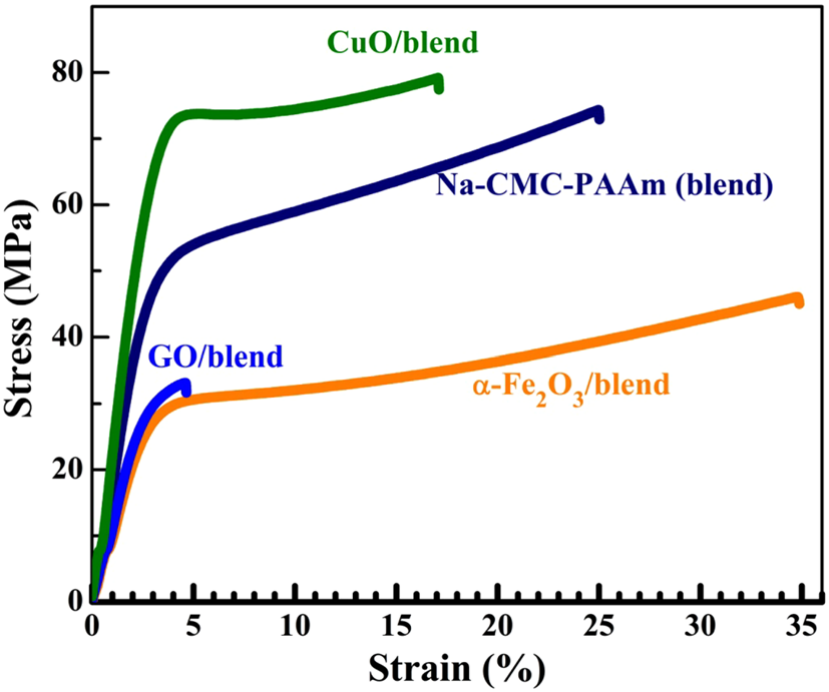
Stress–strain curves of Na-CMC–PAAm blend and nanocomposites.

The mechanical properties of materials can be investigated by analyzing their stress–strain curves. Figure 10 shows the stress–strain curves of Na-CMC–PAAm blend and its nanocomposites. Mechanical parameters such as the tensile modulus (${E}_{\mathrm{t}}$), tensile strength (${\sigma }_{\mathrm{M}}$), stress at break (*σ*_b_), strain at tensile strength (${\varepsilon }_{\mathrm{M}})$, strain at break (*ε*_b_), and toughness of Na-CMC-PAAm blend and nanocomposites are estimated and compered in Table 2. As evident in Fig. 10, the blend and its nanocomposites exhibit similar mechanical performance. However, *α*-Fe_2_O_3_, GO and CuO induce different influences on the mechanical behavior of the Na-CMC–PAAm blend. The addition of *α*-Fe_2_O_3_ and GO leads to lower values of ${E}_{\mathrm{t}},$ ${\sigma }_{\mathrm{M}}$ and *σ*_b_ as compared with the blend, whereas the addition of CuO enhances those values. The observed increase in ${E}_{\mathrm{t}}$ evinces the ability of CuO to promote more stiffness than the blend. Several essential factors influence the nanocomposite strength such as the filler–polymer matrix interfacial adhesion, particle loading and homogenous distribution of filler within the polymer matrix. The compatibility between CuO and the chains of Na-CMC–PAAm blend facilitates the stress transfer from the blend matrix to the stronger phase of CuO, thus enhances the strength of nanocomposite film^62^. In addition, Table 2 showed that all nanocomposites are characterized by toughness (the integrated area under the stress–strain curve) values lower than that of the Na-CMC–PAAm blend, especially the GO/blend sample. Meanwhile, the ${\varepsilon }_{B}$ value of the blend decreases with GO and CuO inclusion, whereas *α*-Fe_2_O_3_ increases it, that is, *α*-Fe_2_O_3_/blend exhibits the highest ductility. In consistent with TGA data, the observed increase in ${\varepsilon }_{B}$ value with adding *α*-Fe_2_O_3_ is related to the highest water content of *α*-Fe_2_O_3_/blend. The addition of GO induces a dramatic reduction in ${\varepsilon }_{B}$ and toughness values of the blend from 25% and 1459.4 MPa to 4.7% and 102.8 MPa, respectively, which could be explained in terms of the high rigidity and limited molecular mobility resulting from the strong interaction between GO fillers with sheet morphology (cf. Fig. 1d) and the blend matrix^63^.

**Table 2:** Mechanical properties of Na-CMC–PAAm (blend) and the blend loaded with α-Fe_2_O_3_, GO and CuO: Tensile modulus (${E}_{\mathrm{t}}$), Tensile strength (${\sigma }_{\mathrm{M}}$), Strain at tensile strength (${\varepsilon }_{\mathrm{M}}$), Stress at break (${\sigma }_{\mathrm{B}}$), Strain at break (${\varepsilon }_{\mathrm{B}}$), and toughness. Each value represents mean ± standard error (SE).

Film composition	${{\varvec{E}}}_{{\varvec{t}}}$(MPa)	${{\varvec{\sigma}}}_{{\varvec{M}}}$(MPa)	${{\varvec{\varepsilon}}}_{{\varvec{M}}}$(*%*)	${{\varvec{\sigma}}}_{{\varvec{B}}}$(MPa)	${{\varvec{\varepsilon}}}_{{\varvec{B}}}$(*%*)	Toughness (MPa)
Na-CMC–PAAm (blend)	1790 ± 66.9	74.4 ± 2.6	25 ± 2.0	72.9 ± 2.8	25 ± 2.0	1459.4 ± 153.0
*α*-Fe_2_O_3_ NP/blend	884 ± 94.7	46.1 ± 5.9	34.8 ± 1.9	45.0 ± 5.3	34.9 ± 1.9	1210.9 ± 214.0
GO NS/blend	1150 ± 24	33.2 ± 3.9	4.6 ± 0.50	31.7 ± 4.7	4.7 ± 0.56	102.8 ± 10.4
CuO NP/blend	2150 ± 11.8	79.2 ± 2.9	17.1 ± 1.0	77.4 ± 2.6	17.1 ± 1.0	1158.3 ± 102.5

### Antifungal properties

**Figure 11:**
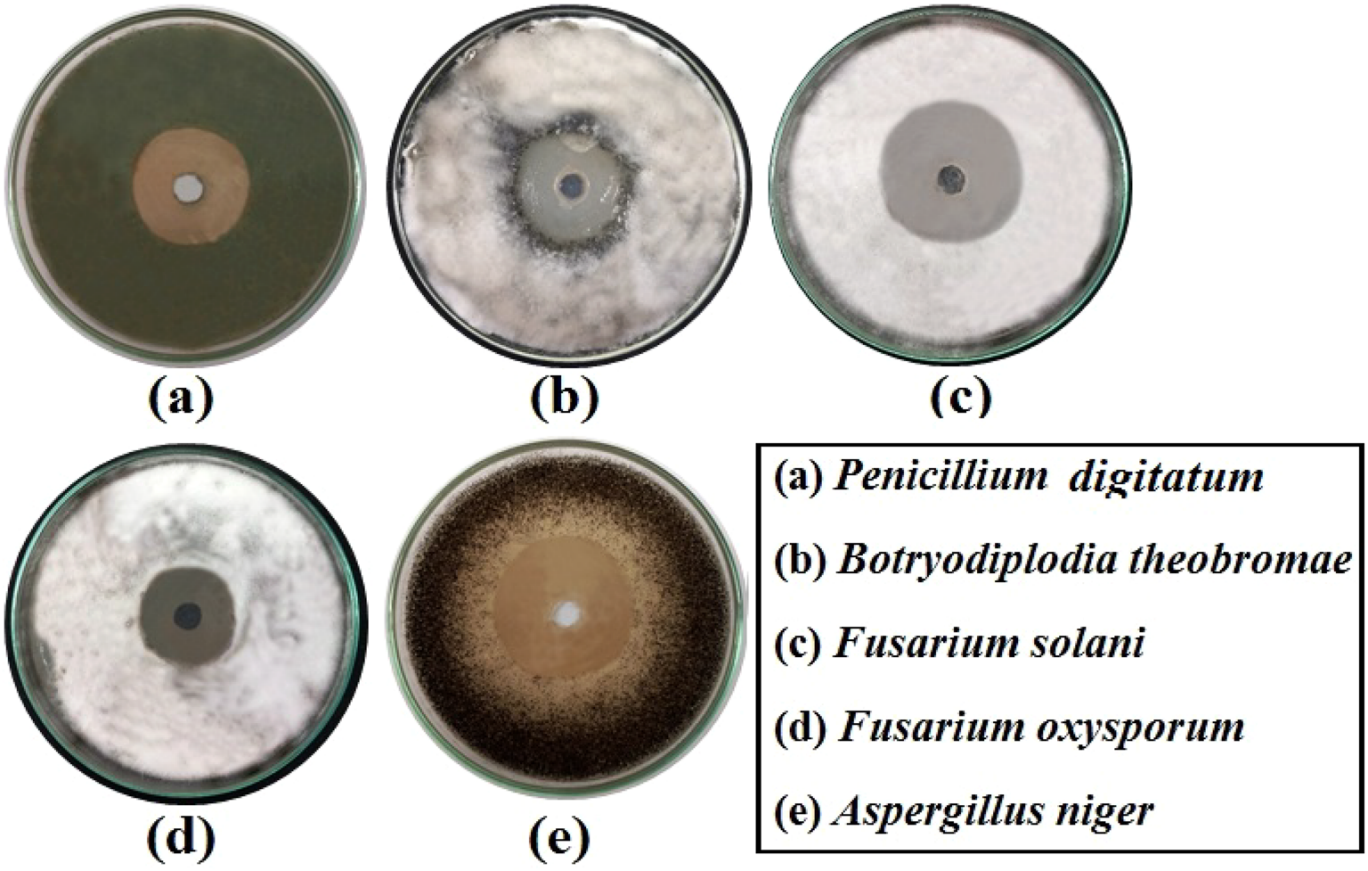
(**a**)-(**e**) Antifungal activity (Zone of inhibition, mm) of the most effective nanocomposite (CuO/Na-CMC–PAAm (blend)) against various phytopathogenic fungi.

In this experiment, antifungal activity of examined nano-composites (Na-CMC–PAAm (blend), GoO/blend, *α*-Fe_2_O_3_/blend, and CuO/blend against some phytopathogenic fungi represented in *P. digitatum, B. theobromae, F. oxysporum, F. solani* and* A. niger* were evaluated using agar well diffusion assay (inhibition zone diameter, mm). The results in Table 3 showed that, all tested nano-composites exception the blend exhibited a significant difference (*p* < 0.0001) in the degrees of antifungal activity against tested phytopathogenic fungal isolates, as indicated by inhibition zone diameters. These data in Table 3 and Fig. 11 showed that CuO/blend and GO/blend were the most effective nano-composites against all tested fungal isolates (mean; 35.00 and 21.73 mm, respectively), followed by *α*-Fe_2_O_3_/blend (13.53 mm). Also, the data revealed that *A. niger* was the most sensitive to all tested nano-composites except the blend, followed by *B. theobromae*, and *F. solani* (mean; 23.08, 20.92 and 20.50, respectively), while *F. oxysporum* and *P. digitatum* were the least sensitive to all tested nano-composites (mean; 6.33 and 17.00 mm, respectively). On the other hand, the blend had no antifungal activity on all tested fungal isolates. Additionally, both GO/blend and *α*-Fe_2_O_3_**/**blend could not inhibit *F. oxysporum* isolate. Moreover, data in Table 3 show that, the highest inhibition zone was recorded for CuO/blend (40.67 mm) against *B. theobromae*, 39.00 mm against *F. solani* and followed by 37.00 and 33.00 mm for *A. niger and P. digitatum,* respectively.

**Table 3:** Evaluation of the antifungal activity of nano-composites against some phytopathogenic fungi.

Fungi	Zone of inhibition (mm)				Mean	LSD_0.05_
	Na-CMC–PAAm (blend)	GO/blend	*α*-Fe_2_O_3_/ blend	CuO/blend		
*P. digitatum*	0.0^d^ ± 0.0	20.67^b^ ± 0.9	14.33^c^ ± 0.7	33.0^a^ ± 1.0	17.00	2.74**
*B. theobromae*	0.0^d^ ± 0.0	31.67^b^ ± 1.2	11.33^c^ ± 0.3	40.67^a^ ± 1.5	20.92	3.34**
*F. solani*	0.0^d^ ± 0.0	26.0^b^ ± 0.6	17.0^c^ ± 0.6	39.0^a^ ± 1.2	20.50	1.99**
*F. oxysporum*	0.0^b^ ± 0.0	0.0^b^ ± 0.0	0.0^b^ ± 0.0	25.33^a^ ± 1.2	6.33	2.08**
*A. niger*	0.0^d^ ± 0.0	30.33^b^ ± 0.3	25.0^c^ ± 0.6	37.0^a^ ± 0.6	23.08	1.88**
Mean	0.00	21.73	13.53	35.00		

Each value represents mean ± 1 standard error. ** indicates differences at and *p* ≤ 0.01 probability level. Means sharing the same letter in each column do not differ significantly at *p* < 0.05 according to LSD test.

The inhibition activity results of demonstrated that the tested nano-composite solutions effectively suppressed the growth and activity of tested phytopathogenic fungi. Based on the results presented in Table 4, CuO/blend represented the highest sensitivity to all examined phytopathogenic fungal strains as evidenced by the MIC and MFC values of the investigated nano-composite solutions, followed by GO/blend and *α*-Fe_2_O_3_/blend, while the blend did not show any inhibition or fungicidal effects against the tested fungal strains. The lowest MIC and MFC values among all nano-composite solutions were recorded for CuO/blend against all fungal strains, which were 7.5/1200/300 and 10/1600/400 against *F. solani*, 10/1600/400 and 10/1600/400 against *B. theobromae*, 10/1600/400 and 12.5/2000/500 against *A. nigar*, 10/1600/400 and 15/2400/600 against *P. digitatum* and 17.5/2800/700 and22.5/3600/900 against *F. oxysporum*, respectively. In contrast, the highest MIC and MFC were 20/3200/800 and 25/4000/1000, respectively against *B. theobromae*, which recorded by *α*-Fe_2_O_3_/blend. As for the blend, no MIC and MFC values recorded against studied fungal strains. Where, the highest sensitive strain for most nano-composite solutions was *F. solani*, followed *by B. theobromae* and *A. nigar*. While the lowest sensitive strain was *F. oxysporum*, which was only affected by CuO/blend nano-composite solution. Briefly, the nano-composite CuO/blend was the most effective on examined fungi, then GO/blend and finally *α*-Fe_2_O_3_/blend, while the blend did not exhibit any effect on the studied fungi.

**Table 4:** MIC and MFC of Na-CMC-PAAm blend, GO/blend, *α*-Fe_2_O_3_/blend/, and CuO/blend against some phytopathogenic fungi.

Fungi	Concentration (μg/ml)							
	Blend		GO/blend		*α*-Fe_2_O_3_/blend		CuO/blend	
	MIC	MFC	MIC	MFC	MIC	MFC	MIC	MFC
*P. digitatum*	–	–	15/2400/600	20/3200/800	17.5/2800/700	22.5/3600/900	10/1600/400	15/2400/600
*B. theobromae*	–	–	12.5/2000/500	15/2400/600	20/3200/800	25/4000/1000	10/1600/400	10/1600/400
*F. solani*	–	–	15/2400/600	17.5/2800/700	17.5/2800/700	20/3200/800	7.5/1200/300	10/1600/400
*F. oxysporum*	–	–	–	–	–	–	17.5/2800/700	22.5/3600/900
*A. nigar*	–	–	15/2400/600	15/2400/600	15/2400/600	20/3200/800	10/1600/400	12.5/2000/500

Similar results were obtained by many researchers^33,64,65^**,** where they reported that, GO and Cu NPs can strongly inhibit the mycelial growth and spore germination of various plant fungal pathogens, such as *Fusarium graminearum, Fusarium poaea and F. oxysporum*.,* Fusarium culmorum* and *A. niger*. In addition, El-Batal and others^66^ showed that the antifungal efficacy of Cu-NPs was greatest against *F. oxysporum*, *Alternaria solani*, and *A. niger*. It has been reported that GO treatment can inhibit *F. graminearum* mainly by enriching the metabolic pathway and histidine metabolism. Moreover, the amount of lipid proteins involved in cell-wall synthesis would be decreased at the protein level, which would affect mycelial cell-wall synthesis^33^. *α*-Fe_2_O_3_ NP have a small size and a large surface-to-volume ratio, so they can strongly adhere to the fungal cell surface. They can also directly enter the cell and cause damage to the cell wall. Fungal inactivation by *α*-Fe_2_O_3_ NP includes a direct contact between NP and cell surfaces, which causes oxidative stress in fungal cells, resulting in cell death^67^. IONPs cause ROS generation represented in Fenton reactions and photocatalysis, which have DNA damaging and genotoxic action^67^, in addition to the ability of metal ions to inhibit protiens and enzyme activity by binding with active groups on proteins such as caboxyl, amino, and mecapto groups^67,68^. Furthermore, the ability of IONP particles to penetrate the microbial cell increases their concentration in the cytoplasm and disrupt the cell wall^67,69^.

## Conclusions

*α*-Fe_2_O_3_ and CuO Nano-plates (NP) and GO nano-sheets of crystallite size in the range of 35–61 nm and wide in the range of 47–92 nm have been prepared and loaded into the Na-CMC–PAAm blend. These nano-fillers were well-dispersed inside the blend and reduced its semicrystalline structure from 22.2 to 17.6*%*. FTIR spectra confirmed the existence of all the reactive functional groups of the blend. The intensity of most peaks were decreased after doping, indicating the complexation and interactions with these nano-fillers. FE-SEM analysis indicates that all the films are homogeneous, crack-free, the two polymers in the blend were well-miscible, and the existence of some particle agglomerated on the film surface loaded with GO NS or CuO NP. EDS analysis confirmed the presence of the dopants with small concentrations inside the blend. The films showed T*%* of about 95%, reduced to 85% after doping with *α*-Fe_2_O_3_ NP or CuO NP and to 54–68*%* after loading GO NS. The *k* spectra suggested the formation of O^2−^ → Cu^2+^ charge transfer in the CuO/blend. CuO significantly reduced the *E*_gi_ and *E*_gd_ transitions from 4.8 and 5.5 eV to 3.6 and 4.1 eV, respectively. GO significantly increased the refractive index of the blend from 1.28 to 2.32. These improvements in the optical features make these combinations suitable for optoelectronic devices such as organic light-emitting diodes and photovoltaic cells. The *ɛ′* value of the blend is in the range of 6.25–12.5, significantly increased to the range of 7–13, 6.75–13.5, and 6.5–13.25 after doping with *α*-Fe_2_O_3_, CuO and GO, respectively. The ${\sigma }_{ac}$ of blend follows the Arrhenius behavior and is equal 21.5 × 10^–4^ S/m, increased to 24 × 10^–4^, 22 × 10^–4^, and 23.5 × 10^–4^ S/m, after loading *α*-Fe_2_O_3_ NP, GO NS and CuO NP, respectively. These 2D nano-fillers can form 3D semiconducting networks inside the blend matrix that facilitate the charge transfer complexes. TGA/DTG analysis showed that the addition of CuO enhances the thermal stability of the blend and reduced its weight loss inferring its strong interaction with the blend chains. The results showed that CuO enhanced the mechanical properties of the blend by improving its tensile modulus, tensile strength and stress at break. On the contrary, the existence of *α*-Fe_2_O_3_ reduces such parameters and increases the strain at break. All the tested nanocomposites in solution form have significant antifungal activities against some phytopathogens (e.g., *P. digitatum, B. theobromae, F. oxysporum, F. solani* and* A. niger*). Specifically, CuO/blend exhibited the highest antifungal activity against all examined fungi, and can be used as a candidate substance in the fields of biological control against pre- and post-harvest fungal diseases.

## Supplementary Information

Supplementary Information.

## Data Availability

The datasets used and/or analysed during the current study available from the corresponding author on reasonable request.

## References

[1] El-Sayed S (2011). DSC, TGA and dielectric properties of carboxymethyl cellulose/polyvinyl alcohol blends. Phys. B.

[2] Hassen A (2012). Influence of Cr_2_O_3_ nanoparticles on the physical properties of polyvinyl alcohol. J. Appl. Phys..

[3] El Sayed AM (2015). Effect of PVA and copper oxide nanoparticles on the structural, optical, and electrical properties of carboxymethyl cellulose films. J. Mater. Sci..

[4] Morsi MA, El-Khodary SA, Rajeh A (2018). Enhancement of the optical, thermal and electrical properties of PEO/PAM: Li-polymer electrolyte films doped with Ag nanoparticles. Phys. B.

[5] Ragab HM, Rajeh A (2020). Structural, thermal, optical and conductive properties of PAM/PVA polymer composite doped with Ag nanoparticles for electrochemical application. J. Mater. Sci. Mat. Electron..

[6] Elashmawi IS, Al-Muntaser AA (2021). Influence of Co_3_O_4_ nanoparticles on the optical, and electrical properties of CMC/PAM polymer: Combined FTIR/DFT study. J. Inorg. Organ. Polym. Mater..

[7] Morsi MA (2022). Structural, optical, mechanical, and dielectric properties studies of carboxymethyl cellulose/polyacrylamide/lithium titanate nanocomposites films as an application in energy storage devices. Polym. Test..

[8] Yalcin M, Tornuk F, Toker OS (2022). Recent advances in the improvement of carboxymethyl cellulose-based edible films. Trends Food Sci. Technol..

[9] Hashmi M (2021). Carboxymethyl cellulose (CMC) based electrospun composite nanofiber mats for food packaging. Polymers.

[10] Zhang H, Zhai D, He Y (2014). Graphene oxide/polyacrylamide/carboxymethyl cellulose sodium nanocomposite hydrogel with enhanced mechanical strength: Preparation, characterization and the swelling behavior. RSC Adv..

[11] Awad S (2020). Characterization, optical, and nanoscale free volume properties of Na-CMC/PAM/CNT nanocomposites. Polym. Adv. Technol..

[12] El-Gamal S, El Sayed AM (2020). Influence of MWCNTs in improving the optical, DC conductivity, and mechanical properties of CMC/PAAM blends. Polym. Eng. Sci..

[13] Virya A, Lian K (2018). Lithium polyacrylate-polyacrylamide blend as polymer electrolytes for solidstate electrochemical capacitors. Electrochem. Commun..

[14] Nascimento DWS (2017). Hybrid Biodegradable hydrogels obtained from nanoclay and carboxymethylcellulose polysaccharide: Hydrophilic, kinetic, spectroscopic and morphological properties. J. Nanosci. Nanotechnol..

[15] Yadava M, Rheea KY, Park SJ (2014). Synthesis and characterization of graphene oxide/carboxymethylcellulose/alginate composite blend films. Carbohydr. Polym..

[16] Alqasem B (2017). The enhancement of the magnetic properties of *α*-Fe_2_O_3_ nanocatalyst using an external magnetic field for the production of green ammonia. Mater. Sci. Eng. B.

[17] Zhang Q (2014). CuO nanostructures: Synthesis, characterization, growth mechanisms, fundamental properties and applications. Prog. Mater. Sci..

[18] Gudarzifar H, Rezvani A, Sabbaghi S (2021). Morphological investigation of graphene oxide/polyacrylamide super-elastic nanocomposite by a solution polymerization process with enhanced rheological property and thermal conductivity. Int. J. Nano Dimens..

[19] Madih K (2022). A facile synthesis of cellulose acetate reinforced graphene oxide nanosheets as proton exchange membranes for fuel cell applications. J. Saudi Chem. Soc..

[20] Jhang J-W (2022). One-pot green reduction and surface decoration of graphene oxide nanosheets with PEGylated chitosan for application in cancer photothermal therapy. J. Taiwan Inst. Chem. Eng..

[21] Khezerlou A (2018). Nanoparticles and their antimicrobial properties against pathogens including bacteria, fungi, parasites and viruses. Microb. Pathog..

[22] Slavin YN, Bach H (2022). Mechanisms of antifungal properties of metal nanoparticles. Nanomaterials.

[23] Dizaj SM (2015). Antimicrobial activity of carbon-based nanoparticles. Adv. Pharm. Bull..

[24] Crisan MC, Teodora M, Lucian M (2021). Copper nanoparticles: Synthesis and characterization, physiology, toxicity and antimicrobial applications. Appl. Sci..

[25] Ezealigo US (2021). Iron oxide nanoparticles in biological systems: Antibacterial and toxicology perspective. JCIS Open.

[26] Brauer VS (2019). Antifungal agents in agriculture: Friends and foes of public health. Biomolecules.

[27] El-Baky NA, Amara AAAF (2021). Recent approaches towards control of fungal diseases in plants: An updated review. J. Fungi.

[28] Perrone GA (2007). Biodiversity of *Aspergillus* species in some important agricultural products. Stud. Mycol..

[29] Soares C, Calado T, Venancio A (2013). Mycotoxin production by *Aspergillus niger* aggregate strains isolated from harvested maize in three Portuguese regions. Revistaiberoamericana Micología..

[30] Covey PA (2014). Multilocus analysis using putative fungal effectors to describe a population of *Fusarium oxysporum* from sugar beet. Phytopathology.

[31] Kazemi M (2021). Green synthesis of colloidal selenium nanoparticles in starch solutions and investigation of their photocatalytic, antimicrobial, and cytotoxicity effects. Bioproc. Biosyst. Eng..

[32] Khatami M (2019). Copper oxide nanoparticles greener synthesis using tea and its antifungal efficiency on *Fusarium solani*. Geomicrobiol. J..

[33] Wang X (2021). Synergistic antifungal activity of graphene oxide and fungicides against Fusarium head blight in vitro and in vivo. Nanomaterials.

[34] Wang X (2014). Evaluation and mechanism of antifungal effects of carbon nanomaterials in controlling plant fungal pathogen. Carbon.

[35] Sawangphruk M (2012). Synthesis and antifungal activity of reduced graphene oxide nanosheets. Carbon.

[36] Ibrahim AMM (2023). Improving the optical, dielectric properties and antimicrobial activity of Chitosan–PEO by GO/MWCNTs: Nanocomposites for energy storage and food packaging applications. Polymer.

[37] Parveen S (2018). Preparation, characterization and antifungal activity of iron oxide nanoparticles. Microb. Pathogen..

[38] Saied E (2022). Mycosynthesis of hematite (*α*-Fe_2_O_3_) nanoparticles using *Aspergillus niger* and their antimicrobial and photocatalytic activities. Bioengineering.

[39] Muhamad II (2013). Characterization and evaluation of antibacterial properties of polyacrylamide-based hydrogel containing magnesium oxide nanoparticles. Int. J. Biol. Biomed. Eng..

[40] Coats AW, Redfern J (1964). Kinetic parameters from thermogravimetric data. Nature.

[41] Yan L (2014). Thermogravimetric study on the pressurized hydropyrolysis kinetics of a lignite coal. Int. J. Hydrogen Energy.

[42] Gonelimali FD (2018). Antimicrobial properties and mechanism of action of some plant extracts against food pathogens and spoilage microorganisms. Front. Microbiol..

[43] Zhang A (2015). Synthesis and antimicrobial activities of acrylamide polymers containing quaternary ammonium salts on bacteria and phytopathogenic fungi. React. Funct. Polym..

[44] Espinel-Ingroff A (2002). Optimal testing conditions for determining MICs and minimum fungicidal concentrations of new and established antifungal agents for uncommon molds: NCCLS collaborative study. J. Clin. Microbiol..

[45] Lee TH, Boey FYC, Khor KA (1995). X-ray diffraction analysis technique for determining the polymer crystallinity in a polyphenylene sulfide composite. Polym. Compos..

[46] Al-Muntaser AA (2022). α-MoO_3_ nanobelts/CMC-PVA nanocomposites: hybrid materials for optoelectronic and dielectric applications. J. Polym. Res..

[47] Abdallah EM (2022). Structural, optical, thermal, and dielectric properties of carboxymethyl cellulose/sodium alginate blend/lithium titanium oxide nanoparticles: Biocomposites for lithium-ion batteries applications. Int. J. Energy Res..

[48] El Fewaty NH, El Sayed AM, Hafez RS (2016). Synthesis, structural and optical properties of tin oxide nanoparticles and its CMC/PEG–PVA nanocomposite films. Polym. Sci. Series A.

[49] Fahmy T (2020). AC conductivity and dielectric relaxation of chitosan/poly(vinyl alcohol) biopolymer polyblend. Bull. Mater. Sci..

[50] El Sayed AM, Mohamd AD (2018). Synthesis, structural, thermal, optical and dielectric properties of chitosan biopolymer; influence of PVP and *α*-Fe_2_O_3_ Nanorods. J. Polym. Res..

[51] Zhang Y-S (2021). Heterogeneous BaTiO_3_@Ag core-shell fibers as fillers for polymer dielectric composites with simultaneously improved dielectric constant and breakdown strength. Compos. Commun..

[52] Mohammed Gh, El Sayed AM (2019). Structural, morphological, optical and dielectric properties of M^3+^/PVA/PEG SPE Films (M = La, Y, Fe or Ir). Polym. Adv. Technol..

[53] El-Bana MS (2018). Preparation and characterization of PbO/carboxymethyl cellulose/polyvinylpyrrolidone nanocomposite films. Polym. Compos..

[54] Elsaeedy HI, Ali HE, Algarni H, Yahia IS (2019). Nonlinear behavior of the current–voltage characteristics for erbiumdoped PVA polymeric composite films. Appl. Phys. A.

[55] Ali HE (2018). Optical spectroscopy and electrical analysis of La^3+^-doped PVA composite films for varistor and optoelectronic applications. J. Mater. Sci. Mat. Electron..

[56] Dueramae I (2020). Properties enhancement of carboxymethyl cellulose with thermo–responsive polymer as solid polymer electrolyte for zinc ion battery. Sci. Rep..

[57] Pettignano A, Charlot A, Fleury E (2019). Solvent-free synthesis of amidated carboxymethyl cellulose derivatives: Effect on the thermal properties. Polymers.

[58] Han F (2013). Thermal properties of carboxymethylcellulose and methyl methacrylate graft copolymers. J. Macromolec. Sci. B.

[59] Voronova MI (2020). Properties of polyacrylamide composites reinforced by cellulose nanocrystals. Heliyon.

[60] Mahmoud WE, Al-Ghamdi AA (2010). The influence of vanadium pentoxide on the structure and dielectric properties of poly(vinyl alcohol). Polym. Int..

[61] Rajeh A, Morsi MA, Elashmawi IS (2019). Enhancement of spectroscopic, thermal, electrical and morphological properties of polyethylene oxide/carboxymethyl cellulose blends: Combined FT-IR/DFT. Vacuum.

[62] Abdolmaleki A, Mallakpour S, Tabebordbar H (2016). Study on morphology, thermal, mechanical and Cd(II) adsorption properties of PVC/α-MnO_2_-stearic acid nanocomposites: Production and application. J. Polym. Res..

[63] Raheel M (2015). Poly (vinyl-alcohol)/GO-MMT nanocomposites: Preparation, structure and properties. Chin. J. Polym. Sci..

[64] Vanathi P, Rajiv P, Sivaraj R (2016). Synthesis and characterization of Eichhornia-mediated copper oxide nanoparticles and assessing their antifungal activity against plant pathogens. Bull. Mater. Sci..

[65] Salem SS (2022). Synthesis of silver nanocomposite based on carboxymethyl cellulose: Antibacterial, antifungal and anticancer activities. Polymers.

[66] El-Batal AI (2020). Penicillium chrysogenum-mediated mycogenic synthesis of copper oxide nanoparticles using gamma rays for in vitro antimicrobial activity against some plant pathogens. J. Clust. Sci..

[67] Gudkov SV (2021). Do iron oxide nanoparticles have significant antibacterial properties?. Antibiotics.

[68] Vega-Jiménez AL (2019). In Vitro Antimicrobial Activity Evaluation Of Metal Oxide Nanoparticles. Nanoemulsions–Properties Fabrications and Applications.

[69] Armijo LM (2020). Antibacterial activity of iron oxide, iron nitride, and tobramycin conjugated nanoparticles against *Pseudomonas aeruginosa* biofilms. J. Nanobiotechnol..

