# Supplementary material for: Influence of α-Fe2O3, CuO and GO 2D nano-fillers on the structure, physical properties and antifungal activity of Na-CMC–PAAm blend
Source: Sci Rep. 2023 Jul 31;13:12358. doi: 10.1038/s41598-023-39056-y (PMC10390538; doi:10.1038/s41598-023-39056-y)
Supplement: Supplementary file 1 — Supplementary Information. [file 41598_2023_39056_MOESM1_ESM.pdf]

Influence of α-Fe_2O_3, CuO and GO 2D Nano-fillers on the Structure, Physical Properties and Antifungal Activity of Na-CMC–PAAm Blend

A. Abou Elfadl[1], Asmaa M.M. Ibrahim[1] , Adel M. El Sayed[1]*, S. Saber[1], Sameh Elnaggar[2], Ibrahim M. Ibrahim[3**]

[1]Physics Department, Faculty of Science, Fayoum University, El Fayoum, 63514, Egypt

[2]Department of Botany, Faculty of Agriculture, Fayoum University, Fayoum 63514, Egypt

[3]Department of Agricultural Microbiology, Faculty of Agriculture, Fayoum University, El Fayoum, 63514, Egypt

Table S1: Film thickness (*d*), the crystallization degree (X_C%), transmission (T%) at 500 nm, indirect and direct optical bandgap, and the index of refraction (*n*) at 500 nm.

Film composition	*d* (mm)	X_C%	T (%) at 500 nm	Bandgap (eV)		*n*
				E_{gi}	E_{gd}	
Na-CMC–PAAm (blend)	0.110	20.22	94.15	4.8	5.5	1.28
α-Fe_2O_3 NP/blend	0.107	18.89	80.56	4.6	5.0	1.64
GO NS/blend	0.112	18.02	54.56	4.4	4.7	2.32
CuO NP/blend	0.115	17.62	83.98	3.6	4.1	1.56

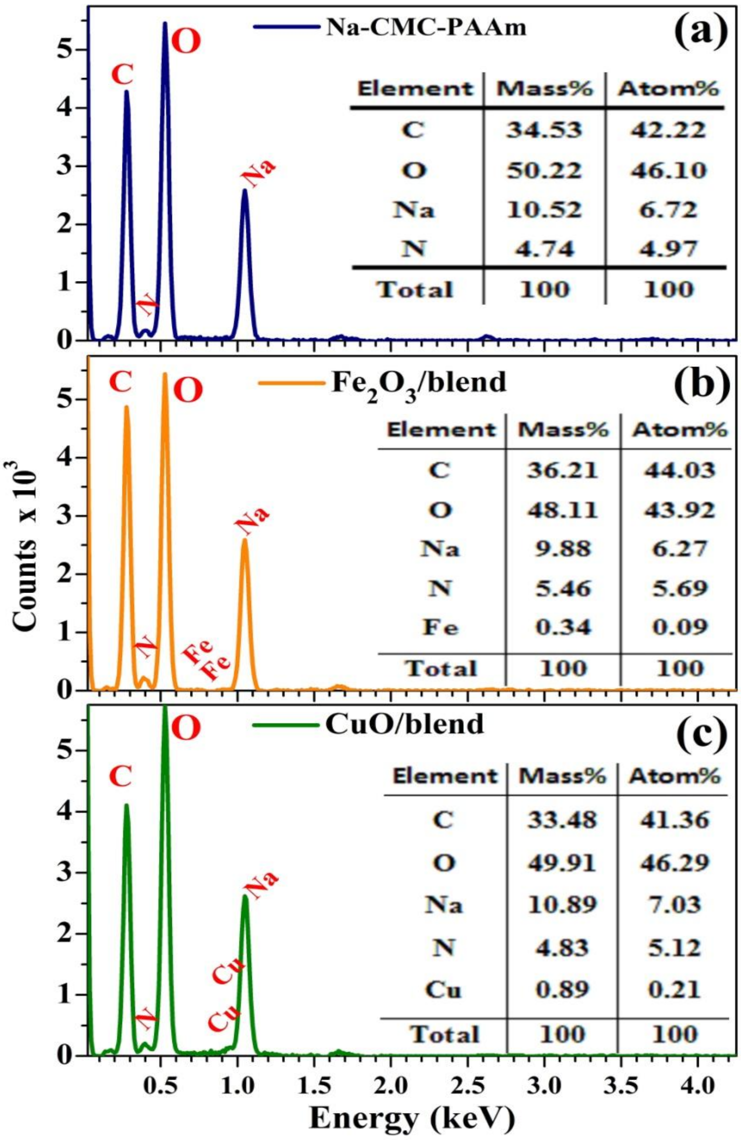

Fig. S1: EDS spectra (chemical analysis) of the pure blend and the blend loaded with α-Fe_2O_3 and CuO NP.

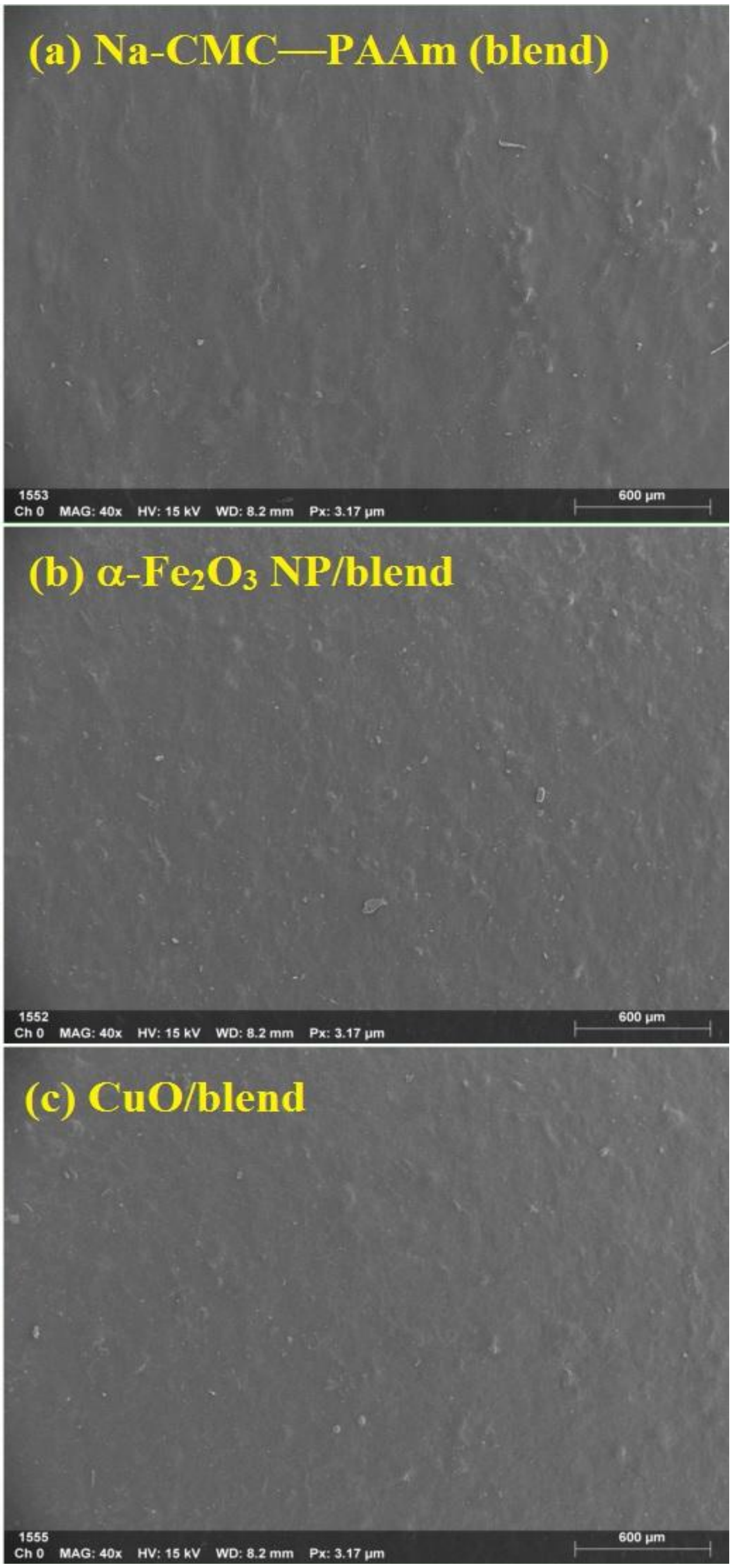

Fig. S2: SEM images (scale bar 600 µm and magnification of 40 X) used to obtain EDS spectra.

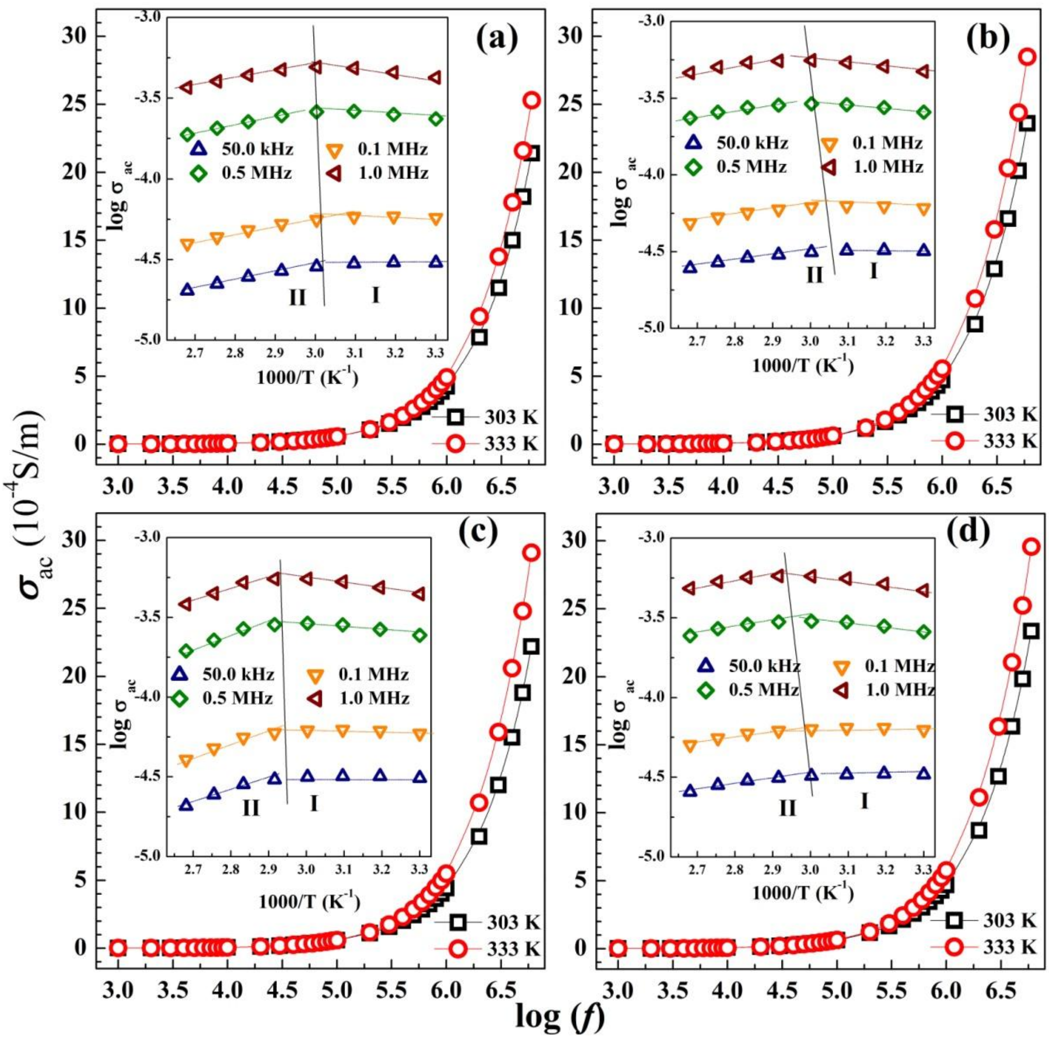

Fig. S3: The dependence of (σ_{ac}) on the applied f for the blend (a), blend loaded with α-Fe_2O_3 NP, GO NS and CuO NP (b-d). The insets show Arrhenius behavior (log σ_{ac} & $\frac{1000}{T}$).
